# Ferroptosis in Rat Lung Tissue during Severe Acute Pancreatitis-Associated Acute Lung Injury: Protection of Qingyi Decoction

**DOI:** 10.1155/2023/5827613

**Published:** 2023-02-11

**Authors:** Peng Ge, Yalan Luo, Qi Yang, Haiyun Wen, Jin Liu, Yibo Zhang, Xuanchi Dong, Guixin Zhang, Caiming Xu, Jing Liu, Zheyi Liu, Hailong Chen

**Affiliations:** ^1^Department of General Surgery, The First Affiliated Hospital of Dalian Medical University, Dalian, Liaoning, China; ^2^Institute (College) of Integrative Medicine, Dalian Medical University, Dalian, Liaoning, China; ^3^Laboratory of Integrative Medicine, The First Affiliated Hospital of Dalian Medical University, Dalian, Liaoning, China; ^4^Department of Traditional Chinese Medicine, The Second Affiliated Hospital of Dalian Medical University, Dalian, Liaoning, China; ^5^Department of Molecular Diagnostics and Experimental Therapeutics, Beckman Research Institute of City of Hope, Biomedical Research Center, Comprehensive Cancer Center, CA, USA; ^6^CAS Key Laboratory of Separation Sciences for Analytical Chemistry, Dalian Institute of Chemical Physics, Chinese Academy of Sciences, Dalian, Liaoning, China

## Abstract

Qingyi decoction (QYD) has anti-inflammatory pharmacological properties and substantial therapeutic benefits on severe acute pancreatitis (SAP) in clinical practice. However, its protective mechanism against SAP-associated acute lung injury (ALI) remains unclear. In this study, we screened the active ingredients of QYD from the perspective of network pharmacology to identify its core targets and signaling pathways against SAP-associated ALI. Rescue experiments were used to determine the relationship between QYD and ferroptosis. Then, metabolomics and 16s rDNA sequencing were used to identify differential metabolites and microbes in lung tissue. Correlation analysis was utilized to explore the relationship between core targets, signaling pathways, metabolic phenotypes, and microbial flora, sorting out the potential molecular network of QYD against SAP-associated lung ALI. Inflammatory damage was caused by SAP in the rat lung. QYD could effectively alleviate lung injury, improve respiratory function, and significantly reduce serum inflammatory factor levels in SAP rats. Network pharmacology and molecular docking identified three key targets: ALDH2, AnxA1, and ICAM-1. Mechanistically, QYD may inhibit ferroptosis by promoting the ALDH2 expression and suppress neutrophil infiltration by blocking the cleavage of intact AnxA1 and downregulating ICAM-1 expression. Ferroptosis activator counteracts the pulmonary protective effect of QYD in SAP rats. In addition, seven significant differential metabolites were identified in lung tissues. QYD relatively improved the lung microbiome's abundance in SAP rats. Further correlation analysis determined the correlation between ferroptosis, differential metabolites, and differential microbes. In this work, the network pharmacology, metabolomics, and 16s rDNA sequencing were integrated to uncover the mechanism of QYD against SAP-associated ALI. This novel integrated method may play an important role in future research on traditional Chinese medicine.

## 1. Introduction

Acute pancreatitis (AP) is a common inflammatory disease of the pancreas, which places a significant financial burden on patients and public health systems due to its rising incidence and hospitalization rates [1]. Mild AP patients have a better prognosis, with a death rate of less than 3%. However, severe acute pancreatitis (SAP) with pancreatic hemorrhage and necrosis could induce systemic inflammatory response syndrome (SIRS) and potentially multiple organ dysfunction syndrome (MODS), causing a mortality rate of 15-35% [1]. Acute lung injury (ALI)/acute respiratory distress syndrome (ARDS) is a frequent SAP consequence and cause of mortality. Elucidation of the mechanism of SAP-associated ALI and the development of effective medications to arrest the disease's malignant progression are urgently needed [2, 3].

Qingyi decoction (QYD) is derived from the traditional Chinese medicine (TCM) formula Da Chai Hu decoction and Da Cheng Qi decoction in “Shanghan Lun.” This TCM formula is often used to treat people with AP because it induces purgation and clears heat and toxic materials [4]. QYD is a robust anti-inflammatory agent that can improve the intestinal barrier damage caused by SAP, microcirculatory disorders, and pulmonary inflammatory response. A recent meta-analysis of 38 clinical trials involving 2254 patients confirmed that QYD dramatically lowered levels of proinflammatory factors, including interleukin- (IL-) 6, tumor necrosis factor-*α* (TNF-*α*), and IL-1*β* while increasing levels of the anti-inflammatory factor IL-10 in patients with AP [5]. However, the fundamental mechanism of QYD for SAP and associated organ injury is yet unknown.

An emerging discipline called network pharmacology (NP) is frequently utilized to reveal the molecular mechanisms of herbal formulas for treating diseases. NP could anticipate the active ingredients and targets of herbal formulas for treating conditions and build ingredient-target networks [6]. Through a thorough investigation of metabolite alterations before and after illness onset, metabolomics directly reflects the disease phenotype at the functional level. Currently, metabolomic methods are frequently used in the research of ALI/ARDS and are crucial for identifying biomarkers and assessing medication effectiveness [7]. Moreover, the alterations of the lung microbiome before and after illness have been reported recently and have gained increasing attention. Several studies have found that the lung microbiome is not only influenced by host illness but also plays a role in disease progression and severity via eliciting host immune responses [8, 9]. However, changes in the lung microbiota of SAP-associated ALI have not been discovered.

Through a comprehensive investigation, the molecular mechanisms by which QYD intervenes in SAP-associated ALI have been determined in this work. We first used a network pharmacology approach to screen potential targets of QYD, which determined the central role of ferroptosis in SAP-associated ALI. In exploring the effect of QYD on ferroptosis, we further combined metabolomics and 16s rDNA sequencing to analyze the correlation of ferroptosis-related markers with differential metabolites and microbes. To sum up, we have presented a new paradigm and provided a novel idea for research into TCM.

## 2. Materials and Methods

### 2.1. Preparation of Qingyi Decoction

The herbal material of QYD is resourced from the First Affiliated Hospital of Dalian Medical University (Dalian, Liaoning, China). QYD consists of *Bupleurum chinense* DC (Chai Hu in Chinese, 15 g), *Corydalis yanhusuo* (Yan Hu Suo in Chinese, 15 g), *Scutellaria baicalensis* Georgi (Huang Qin in Chinese, 12 g), *Gardenia jasminoides* J. Ellis (Zhi Zi in Chinese, 15 g), Paeonia lactiflora Pall. (Bai Shao in Chinese, 15 g), Rheum officinale Baill. (Da Huang in Chinese, 20 g), *Aucklandia costus* Falc. (Mu Xiang in Chinese, 15 g), and Na_2_SO_4_·10 H_2_O (Mang Xiao in Chinese, 10 g). Professor Aijing Leng certified the authenticity of all the herbs. The QYD extract (1 g crude herb/ml) was made according to the reported methods. Briefly, the herbs are weighed, combined with 10 times their weight in water, steeped for 0.5 h, boiled for 1 h, and filtered while still hot. The residue is boiled again in 8 times the quantity of water used for the herbs and decocted for 0.5 h, and then, the *Rheum officinale* Baill. is added and simmered for an additional 0.5 h. Filter when hot, add Na_2_SO_4_·10 H_2_O, blend the two filtrates, concentrate to 1 g/ml, sterile package, and store at 4°C for future use. The chemical profiles of QYD mapped using ultraperformance liquid chromatography coupled with quadrupole time-of-flight mass spectrometry are presented in the previous study [10].

### 2.2. Reagents

Antibodies against ALDH2 (A11500), AnxA1 (A1118), ICAM-1 (A5597), GPX4 (A11243), nuclear factor-*κ*B p65 (p65, A16271), *β*-actin (AC026), and horseradish peroxidase- (HRP-) conjugated anti-rabbit IgG antibody (AS014) were purchased from ABclonal, Inc. (Wuhan, China). Antibodies against phosphorylated p65 (Ser 536, p-p65, bs-0982R) and 8-hydroxy-2′-deoxyguanosine (8-OHdG, bs-1278R) were purchased from Bioss (Beijing, China). Antibodies against SLC7A11 (DF12509), NCOA4 (DF4255), and FTH1 (DF6278) were purchased from Affinity (Liyang, China). Rat tumor necrosis factor-*α* (TNF-*α*, E-EL-R2856c) and interleukin-6 (IL-6, E-EL-R0015c) ELISA kits were obtained from Elabscience Biotechnology Co., Ltd. (Wuhan, China). Glutathione (GSH) assay kit (A006-2-1), amylase assay kit (C016-1-1), myeloperoxidase (MPO) assay kit (A044-1-1), malondialdehyde (MDA) assay kit (A003-1-2), and tissue Fe^2+^ assay kit (A039-2-1) were obtained from Nanjing Jiancheng Bioengineering Institute (Nanjing, China).

### 2.3. Animals and Treatment

All animal experiments were conducted by the People's Republic of China Legislation Regarding the Use and Care of Laboratory Animals and approved by the Committee for Research and Animal Ethics of Dalian Medical University, Dalian, China (approved number: AEE19003). Sprague-Dawley (SD) male rats weighing 180-220 g (6-8 weeks old) were provided by the Experimental Animal Center of Dalian Medical University. All animals were kept in ventilated caging systems at 22.0 ± 2.0°C, with a 12 h light-dark cycle, allowed free access to standard rat chow and water *ad libitum*. Rats were acclimated to the housing condition for one week before experiments. Firstly, 60 rats were randomly assigned to four groups (*n* = 15): a blank control group (the CON group), a severe acute pancreatitis model group (the SAP group), a severe acute pancreatitis model+Qingyi decoction-treated group (the SAP+QYD group), and a blank control+Qingyi decoction-treated group (the CON+QYD group). Rats in the SAP group were retrograde-injected into the biliopancreatic duct with 5.0% sodium taurocholate (STC, 50 mg/kg) as reported previously [11], and the same volume of sterile saline was used in the CON group. Rats in the SAP+QYD and CON+QYD groups were administered Qingyi decoction (10 g/kg, crude drug volume: 1 g/ml) via gastric perfusion 0.5 h before and 12 h after administration of STC and sterile saline. Secondly, 30 SD male rats were then separated into four groups: CON (*n* = 6), SAP (*n* = 8), SAP+QYD (*n* = 8), and SAP+QYD+erastin (*n* = 8). The CON, SAP, and SAP+QYD groups were handled as previously stated. In the SAP+QYD+erastin (Era, ferroptosis activator, MCE) group, QYD was administered by gavage 0.5 h before and 12 h after STC administration, while Era (10 *μ*mol/kg [12]) was administered intraperitoneally 0.5 h after STC administration. Rats were sacrificed after 24 h of STC administration. The pancreas, lung tissues, and blood from each rat were collected and stored at -80°C for subsequent experiments.

### 2.4. Network Pharmacology Analysis

The TCMSP (https://tcmsp-e.com/), TCMIP (http://www.tcmip.cn/), and previous chemical profiles were used to screen the active ingredients of QYD (no active ingredient was detected for Na_2_SO_4_·10 H_2_O), and the screening conditions were referenced to the rules proposed by Lipinski [13]. The 2D and 3D structures of the candidate ingredients were queried using the PubChem (https://pubchem.ncbi.nlm.nih.gov/) database and compared with the data obtained by TCMSP and TCMIP to confirm the final molecular structures. The TCMSP, TCMIP, DrugBank (https://go.drugbank.com/), and SwissTargetPrediction (http://www.swisstargetprediction.ch/) databases were then used to predict the targets of action of the candidate ingredients. Target names were corrected using the UniProt database (https://www.uniprot.org/). In summary, 225 ingredients and 514 potential targets for QYD were identified (Supplementary File S1).

Fold change (FC) > 1.2 or FC < 0.83 with *P* value < 0.05 was used as the primary criterion for differentially expressed proteins (DEPs) [14]. Converting DEPs' names to gene names using the UniProt database. Volcano plots were drawn to show the screening results. The DAVID database (https://david.ncifcrf.gov/) was utilized for the Gene Ontology (GO) and KEGG pathway enrichment analyses. Target names were corrected using the UniProt database, and 3D structures of target proteins were obtained from the AlphaFold database (https://alphafold.ebi.ac.uk/). Cytoscape 3.8.2 software was used to construct the ingredient-target network. The operation procedure was as follows: (1) preprocess the data to generate two original files, network, and type, respectively; (2) import the original data into the software and adjust the edges, nodes, and colors according to the type classification; (3) set the size of each node according to the degree value to highlight the targets and ingredients.

### 2.5. Molecular Docking

The ingredients and targets were imported into Discovery Studio (DS) software and then pretreated with dehydration and hydrogenation and selected as receptor and ligand, respectively. Precise docking was performed using the CDOCKER module of DS software, and the ligand-receptor binding mode was analyzed. CDOCKER is a semiflexible docking program based on the CHARMM force field and determines the binding activity by calculating the ligand-receptor interaction energy [15]. In general, lower ligand-receptor interaction energies in CDOCKER docking represent better binding of ingredients and targets during docking.

### 2.6. Histopathological Analysis

Isolated pancreatic and pulmonary specimens were fixed in 4% paraformaldehyde for 24 h, paraffin-embedded, and sectioned (4 *μ*m). The sections were stained with hematoxylin and eosin (HE). The pathological score of stained sections was conducted in a blinded fashion referred to previous reports [16, 17]. Three parameters (inflammatory cell infiltration, vacuolization, and acinar necrosis) were utilized to determine the pancreatic histological score. The pulmonary histological score was based on three parameters: inflammatory cell infiltration, hemorrhage, and alveolar septal thickening. Each of the individual parameter was graded from 0 to 3. The total score for the three parameters was calculated. The specimens of at least three rats from each group were evaluated.

### 2.7. Measurement of Serum Amylase

Serum was extracted from rat blood by centrifuging at 3000 rpm for 10 min. Serum amylase activity was measured using a commercial amylase assay kit. Briefly, the serum sample was added with a known concentration and excess substrate (amylum). The amylase in the sample hydrolyzed the amylum, and then, iodine was added to react with the unhydrolyzed amylum, generating some blue compounds. The amount of hydrolyzed amylum could be deduced from the absorbance of blue compounds at 660 nm, and thus, the amylase activity could be calculated. The unit was expressed as U/l.

### 2.8. Assessment of Serum Levels of Inflammatory Factors

Concentrations of inflammatory cytokines (IL-6 and TNF-*α*) in rat serum were measured by commercial ELISA kits following to manufacturer's instructions.

### 2.9. Lung Wet/Dry Weight Ratio

The lung wet/dry weight (W/D) ratio reflects pulmonary edema and congestion. The inferior lobe of the rat's right lung was cut out, and the fluid on the lung surface was wiped using filter paper. Lung tissue was weighed immediately for its wet weight and again after drying at 80°C for 48 h to determine its dry weight. The wet weight was divided by the dry weight to obtain the lung W/D ratio.

### 2.10. MPO Activity

MPO activity in lung tissue homogenate was measured as an indication of pulmonary neutrophil infiltration via a commercial assay kit provided by Nanjing Jiancheng Bioengineering Institute (Nanjing, China). The unit was expressed as U/g.

### 2.11. Fe^2+^, GSH, and MDA Measurements

The concentration of Fe^2+^, GSH, and MDA in lung tissue represents pulmonary ferroptosis to some extent. The Fe^2+^ assay kit, GSH assay kit, and MDA assay kit, purchased from Nanjing Jiancheng Bioengineering Institute, were used to detect Fe^2+^, GSH, and MDA levels in lung tissues strictly with the manufacturer's instructions.

### 2.12. Quantitative Real-Time PCR

RNA extraction from rat lung tissue in each group was performed with *RNAex Pro* Reagent (AG21102, Accurate Biology, Changsha, China). The obtained single-stranded mRNA was reverse transcribed into cDNA using the *Evo M-MLV* Reverse Transcription Mix Kit (AG11728, Accurate Biology). Synthesized cDNA was amplified by SYBR® Green Premix *Pro Taq* HS qPCR Kit II (AG11702, Accurate Biology). All primers used are listed in Supplementary File S2. The expression of the target gene relative to the house-keeping gene GAPDH was quantified by the 2^-*ΔΔ*CT^ method [18]. Three independent samples were analyzed in each group.

### 2.13. Western Blotting

The Protein Extraction Kit and the BCA Protein Quantitation Kit (KGP10100, KGP903, KeyGen Biotech, Nanjing, China) were used to achieve protein extraction and quantification of rat lung tissue. Protein samples were separated via 10%-12% sodium dodecyl sulfate-polyacrylamide gel electrophoresis and electrotransferred onto 0.22 *μ*m polyvinylidene fluoride membranes. The membranes were blocked with 5% bovine serum albumin solution for 2 h at room temperature and then incubated overnight at 4°C with the following primary antibodies: ALDH2 (1 : 1000), GPX4 (1 : 1000), p65 (1 : 1000), p-p65 (1 : 500), AnxA1 (1 : 1000), ICAM-1 (1 : 1000), and *β*-actin (1 : 50 000). After TBST solution rinsing, the membranes were incubated with HRP-conjugated secondary antibodies for 2 h at room temperature. Protein levels were visualized and quantified using a chemiluminescence system (Tanon-4800, Shanghai, China) and ImageJ software.

### 2.14. TdT-Mediated dUTP Nick End Labeling (TUNEL) Assay

Sections of lung tissue were stained using a TUNEL kit (Roche, Basel, Switzerland). Follow the directions provided by the supplier of the reagent to the letter. Briefly, paraffin-embedded lung tissue was sectioned at a 5 *μ*m thickness for dewaxing and hydration and subsequent antigen repair. Sections were blocked using the serum for 30 minutes, and then, appropriate amounts of TdT and dUTP (1 : 10 mix) were added and incubated overnight at 4°C in a moist box. 4′,6-Diamidino-2-phenylindole (DAPI) was used to restain cell nuclei and micrographs were taken. TUNEL − positive cell rate = (number of TUNEL − positive cells/total number of cells) × 100%.

### 2.15. Immunofluorescence

Paraffin-embedded lung tissue was sectioned at a 5 *μ*m thickness for dewaxing, hydration, and repair. Sections were blocked using 10% goat serum and incubated at 37°C for 0.5 h. The sections were sequentially incubated with diluted primary antibody (incubated at 4°C overnight) and secondary antibody (incubated at 37°C for 1 h). DAPI working solution was added dropwise to the tissue sections and incubated for 5 min at room temperature before washing 3 times with PBS. The liquid on the sections was shaken off, an antifluorescent agent was added, and the sections were sealed and kept at 4°C and out of the light. The sections were observed using a fluorescent microscope.

### 2.16. Metabolomic Analysis

An aliquot of 10 mg lung tissue was placed into a centrifuge tube, added with 1 ml of extraction reagent containing internal standard [19], vortexed, and homogenized thoroughly. The homogenate was sonicated in an ice-water bath for 10 min and incubated at -20°C for 1 h to fully precipitate the proteins. The mixture was dispensed by centrifugation at 14000 g for 10 min at 4°C. Take 50 *μ*l of supernatant from each tube and mix to obtain quality control (QC) samples. Lyophilize all samples to be tested. The lyophilized powder was redissolved in 100 *μ*l of methanol/water (4/1) and then vortex-mixing for 2 min. The supernatant was obtained by centrifugation at 14000 g for 10 min at 4°C. 60 *μ*l of supernatant was taken from each tube for analysis. Chromatographic-mass spectrometry conditions were referenced to previously reported methods [20]. Samples were separated using an Acquity UPLC system and a Waters Acquity BEH C8 column (100 mm × 2.1 mm and 1.7 *μ*m). Details of the analytical conditions and data preprocessing are provided in Supplementary File S3. Metabolites were identified and annotated using the mzCloud (https://www.mzcloud.org/),the KEGG (https://www.genome.jp/kegg/), and the HMDB (https://hmdb.ca/) databases. Metabolites with variable importance in the projection (VIP) > 1, FC > 1.2 or FC < 0.83, and *P* value < 0.05 were considered significant. MetaboAnalyst 5.0 database (http://www.metaboanalyst.ca/) was used for partial least squares discriminant analysis (PLS-DA), orthogonal partial least squares discriminant analysis (OPLS-DA), and metabolic pathway analysis.

### 2.17. 5R 16s rDNA Sequencing Analysis

Samples were collected, processed, and sequenced, referring to previous articles. 50 mg of lung tissue samples was extracted using the CTAB method. 16s rRNA gene amplification and sequencing were done by amplifying 5 regions on the 16s rRNA gene in the multiplex. The libraries were sequenced on Illumina NovaSeq 6000 system. Reads were demultiplexed per sample, filtered, and aligned to each of the five amplified regions based on the primers' sequences. The Short MUltiple Regions Framework (SMURF) method was applied to combine read counts from the five regions into coherent profiling results solving a maximum likelihood problem. For this purpose, we referred to the GreenGenes database (updated version from May 2013). Differences in microbial communities were analyzed using an online website (https://www.omicstudio.cn/). Alpha diversity was performed using the Chao1, Shannon, and Simpson indices. Differences in lung microbiota composition between the CON and SAP groups and between the SAP and QYD groups were uncovered using the linear discriminant analysis effect size (LEfSe). Taxa having an LDA score of >3.0 were deemed to be significant. We utilized PICRUSt2, which stands for Phylogenetic Investigation of Communities by Reconstruction of Unobserved States 2, to infer metabolic function from 16s rDNA sequences.

### 2.18. Statistical Analysis

All experimental data were presented as mean ± standard deviation (SD). One-way analysis of variance (ANOVA) was performed for comparisons among multiple groups. The post hoc test was estimated using Fisher's least square difference (LSD, if homogeneity of variance was satisfied) or Tamhane (if homogeneity of variance was not satisfied). Correlation analysis of ferroptosis-related indicators, differentially metabolites, and microbiota was performed using the Spearman rank correlation analysis. *P* value < 0.05 indicates statistical significance (^∗^*P* < 0.05, ^∗∗^*P* < 0.01, and ^∗∗∗^*P* < 0.001). Statistical analysis and visualization were performed using SPSS 24.0 and GraphPad Prism 8.2.

## 3. Results

### 3.1. QYD Alleviated SAP-Associated ALI

**Figure 1:**
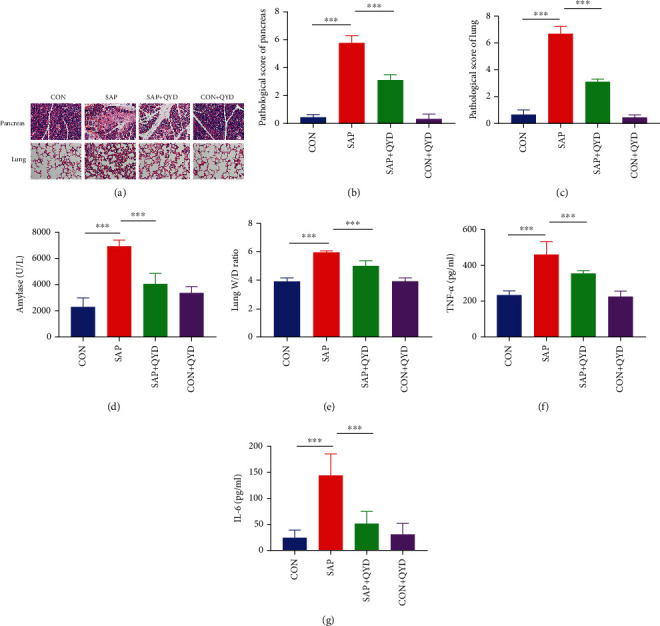
Protective effect of QYD on pancreatic and pulmonary tissue injury in rats with SAP. (a) Representative images of HE staining of the pancreas (upper) and lung tissues (lower) of rats in each group (scale bar, 100 *μ*m). (b, c) Histopathological scores of pancreas and lung tissues of rats in each group. (d) Serum amylase levels of rats in each group. (e) The lung W/D ratio was used to evaluate pulmonary edema in each group of rats. (f, g) Serum levels of inflammatory factors TNF-*α* and IL-6 of rats in each group. Data are presented as representative images or as the mean ± SD of each group of rats (*n* = 6 per group) from at least three separate experiments. ^∗∗∗^*P* < 0.001.

Rats injected with STC showed histopathological characteristics of SAP, including diffuse necrosis of acinar cells, vacuolization, inflammatory cell infiltration, and hemorrhage. Meanwhile, secondary ALI was observed in rats with SAP induced by STC. Lung tissue destruction was evident in rats in the SAP group, such as alveolar and interstitial infiltration of inflammatory cells, congestion, and alveolar septal thickening (Figure 1(a)). After the gavage of QYD, the histopathological damage to rat pancreas and lung was significantly improved, and the pathological score was reduced compared with the SAP group (Figures 1(b) and 1(c)). Serum amylase is a biomarker with high accuracy, specificity, and sensitivity in the early diagnosis of acute pancreatitis. The lung wet/dry weight ratio is an indicator to evaluate pulmonary edema and congestion. Consistent with histological findings, QYD significantly inhibited the increase of serum amylase (Figure 1(d)) and lung wet/dry weight ratio (Figure 1(e)) in SAP rats. The accumulation of circulating inflammatory mediators is an essential bridge in the progression of the pancreas from local inflammation to SIRS and distal organ damage. Serum levels of TNF-*α* and IL-6 were higher in the SAP group than in the CON group, as shown in Figures 1(f) and 1(g), and QYD considerably reduced these inflammatory factor levels in the SAP group. These results suggest that QYD could improve pancreatic inflammation and associated ALI during SAP.

### 3.2. Target and Mechanism Analysis of QYD against SAP-Associated ALI

**Figure 2:**
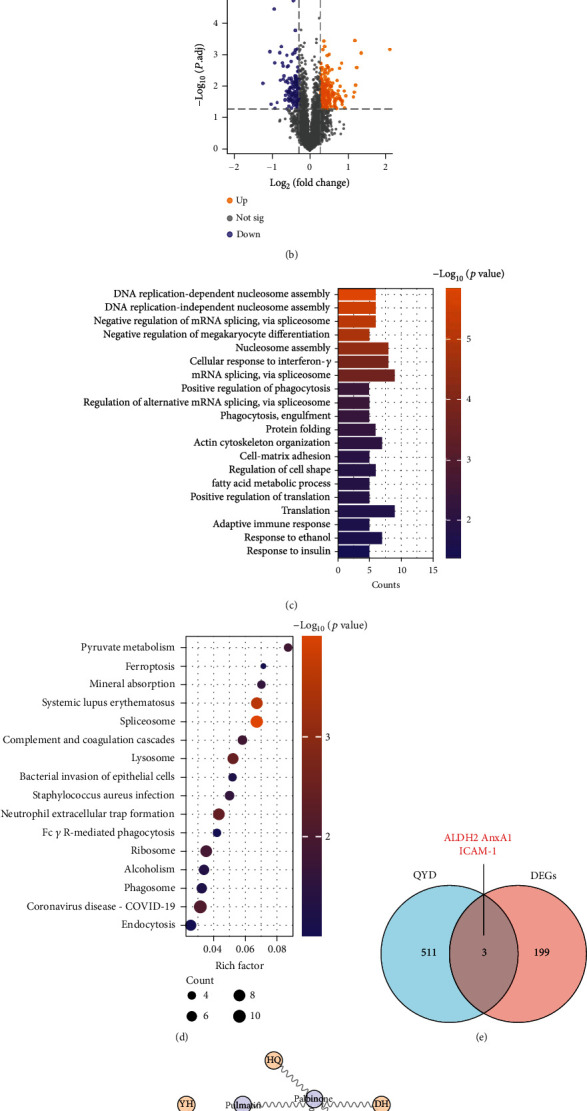
Determination of core targets of QYD against SAP-associated ALI. (a) Volcano plot showing DEPs between the SAP and CON groups. (b) Volcano plot showing DEPs between the QYD and SAP groups. (c) GO enrichment analysis of 202 overlapped DEGs in the term of biological process. (d) Pathway annotation of 202 overlapped DEGs by KEGG terms. (e) Venn diagram between the 202 overlapped DEGs and the 514 drug targets of QYD. The integrated analysis of proteomic data and network pharmacology results yielded three core targets of QYD for SAP-associated ALI: ALDH2, AnxA1, and ICAM-1. (f) Construction of a drug-active ingredient-target network based on the three core targets obtained. Yellow dots represented each herb of QYD; purple dots represented the active ingredients of QYD; red dots represented the target proteins.

Previous proteomic data were used to preliminarily screen possible targets for QYD intervention in SAP-associated ALI [14]. As shown in Figures 2(a) and 2(b), 731 differential expression proteins (DEPs) occurred between the SAP and CON groups based on the screening criteria stated in Materials and Methods, while 382 DEPs existed between the QYD and SAP groups. Among these, 202 differentially expressed genes (DEGs) (Supplementary File S1) with overlap were discovered and used for functional enrichment analysis. The GO biological process terms of the overlapped DEGs were mainly involved in DNA replication-dependent nucleosome assembly, negative regulation of megakaryocyte differentiation, nucleosome assembly, cellular response to interferon-gamma, cell-matrix adhesion, fatty acid metabolic process, adaptive immune response, and response to ethanol (Figure 2(c)). KEGG enrichment analysis revealed that the overlapped DEGs were more enriched in signaling pathways such as ferroptosis, pyruvate metabolism, and neutrophil extracellular trap (NET) formation (Figure 2(d)). According to these data, QYD may exert a protective effect by regulating the expression patterns of genes related to lipid metabolism, ferroptosis, and neutrophil function.

### 3.3. Ingredient-Target Network Construction and Molecular Docking

**Figure 3:**
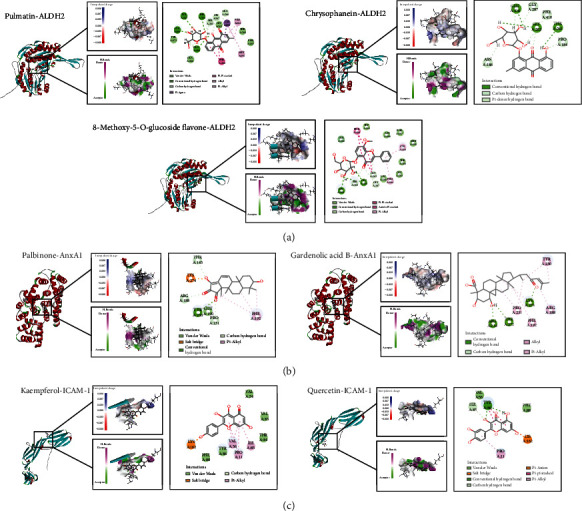
Interactions between ingredients and targets. (a) Molecular docking interaction of pulmatin, chrysophanein, and 8-methoxy-5-O-glucoside flavone with ALDH2. (b) Molecular docking interaction of palbinone and gardenolic acid B with AnxA1. (c) Molecular docking interaction of quercetin and kaempferol with ICAM-1. The left part of each molecular docking result showed the structure of the ingredient-target complex after docking; the middle part showed the interaction space of interpolated charge and hydrogen bonds (acceptor in green and donor in pink) between the ingredient and the target; and the right part showed the two-dimensional diagram of interaction sites between the ingredient and the target.

To further certain the core targets of QYD against SAP-associated ALI, we searched the drug targets of QYD using network pharmacology and performed a secondary screening in combination with proteomics data. As depicted in Figure 2(e), network pharmacology identified 514 drug targets (Supplementary File S1) of QYD intersected with 202 DEGs to generate three core targets of QYD in the treatment of SAP-associated ALI: acetaldehyde dehydrogenase 2 (ALDH2), annexin A1 (AnxA1), and intercellular cell adhesion molecule-1 (ICAM-1). Based on these three intersecting targets, the active ingredient was identified in reverse, and the drug-active ingredient-target network was established (Figure 2(f)). Next, the structures of core targets and corresponding active ingredients were imported into the DS software for CDOCKER docking.  Figure 3(a) shows the binding pattern of ALDH2 to several ingredients, such as pulmatin, chrysophanein, and 8-methoxy-5-O-glucoside flavone.  Figure 3(b) shows the binding pattern of AnxA1 to ingredients such as palbinone and gardenolic acid B.  Figure 3(c) shows the binding pattern between ICAM-1 and ingredients such as kaempferol and quercetin. The CDOCKER interaction energy of all ingredients and proteins during molecular docking is shown in Supplementary File S4. According to the findings, each active ingredient formed several strong hydrogen bonds with its core target and had low binding energies. In a word, QYD may be able to directly target and affect the activity of the core target since its active ingredients have been shown to have a high affinity for the core target in the study.

### 3.4. QYD Inhibits Ferroptosis and Apoptosis by Activating ALDH2

**Figure 4:**
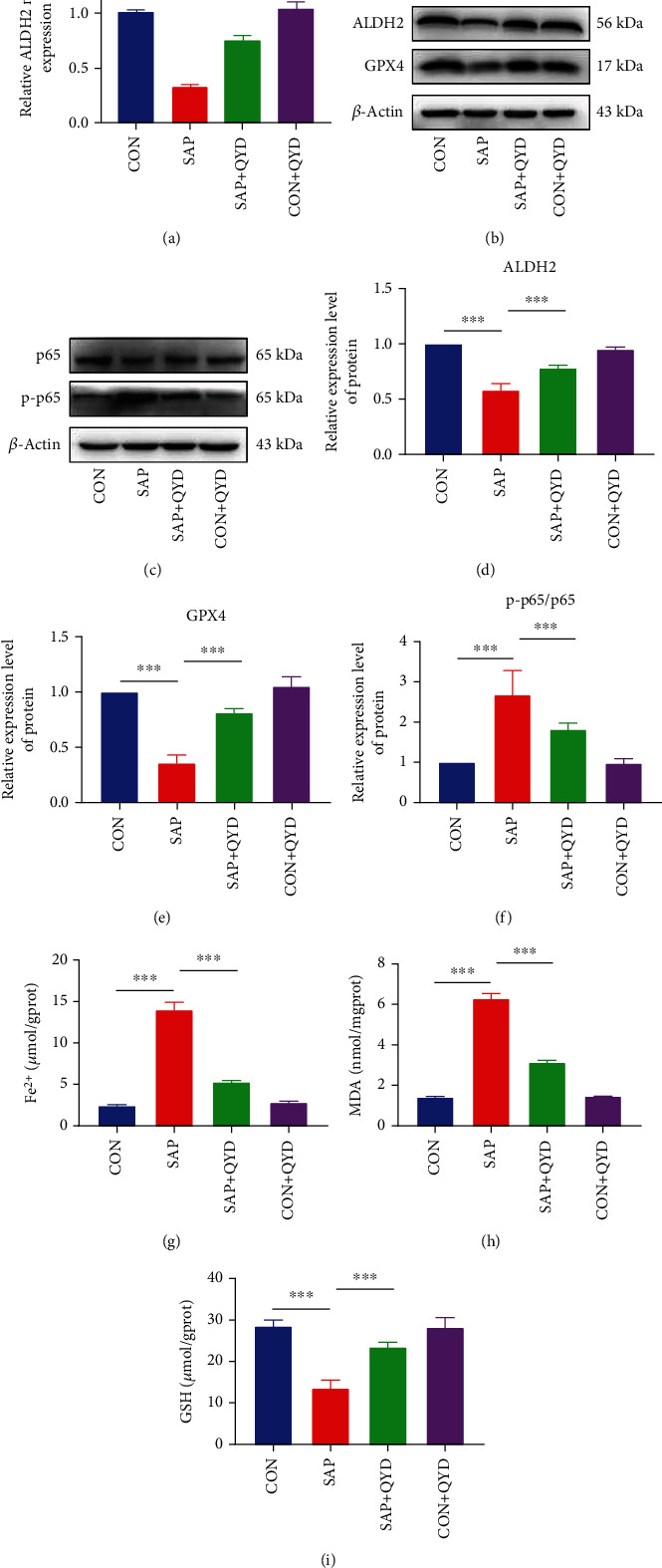
QYD enhancing ALDH2 activity protects against ferroptosis in SAP-associated ALI. (a) qRT-PCR was performed to assess the mRNA expression level of ALDH2 in the rat lung tissue of each group. GAPDH was used as the reference gene. (b, c) Western blotting was conducted to evaluate the protein expression levels of ALDH2, GPX4, p65, and p-p65 in the lung tissue of rats. *β*-Actin was used as a loading control. (d–f) Semiquantification of protein expression of ALDH2, GPX4, and p-p65 using histograms. (g) Measurement of Fe^2+^ in rat lung tissue of each group served as an iron metabolism indicator. (h) MDA concentration in the rat lung tissue of each group was measured as an indicator of lipid peroxidation. (i) GSH levels in rat lung tissue of each group were monitored to reflect antioxidant capacity. Data are presented as representative images or as the mean ± SD of each group of rats (*n* = 6 per group) from at least three separate experiments. ^∗∗∗^*P* < 0.001.

To validate the core targets acquired from the integrative analysis of proteomics and network pharmacology, qRT-PCR and western blotting were first performed to detect the expression levels of ALDH2 in rat lung tissues. ALDH2, a mitochondrial aldehyde dehydrogenase, has recently been reported to play a protective role in various inflammatory diseases by regulating lipid peroxidation and ferroptosis [21, 22]. As shown in Figures 4(a) and 4(b), lung tissue of rats in the SAP group exhibited lower levels of ALDH2 mRNA and protein than those in the CON group, while QYD significantly promoted the transcription and translation of ALDH2. In addition, the apoptotic rate was much higher in the lung tissues of SAP rats than in normal rats. Still, apoptosis induced by SAP was significantly decreased by QYD therapy (Supplementary File S5). Glutathione peroxidase 4 (GPX4) is a key regulator of ferroptosis, preventing cellular ferroptosis by eliminating peroxides, reducing reactive oxygen species (ROS) accumulation, and inhibiting NF-*κ*B activation [23]. As indicated in Figures 4(c)–4(f), GPX4 was decreased, and p-p65 increased in lung tissues of rats in the SAP group compared to those in the CON group. However, GPX4 protein expression was elevated compared with that of the SAP group following the administration of QYD. In contrast, the level of p-p65 protein expression could be reduced by QYD administration. In addition, solute carrier family 7 member 11 (SLC7A11), ferritin heavy chain 1 (FTH1), and nuclear receptor coactivator 4 (NCOA4) were ferroptosis marker genes. SLC7A11 and FTH1 protein expressions were dramatically downregulated, and NCOA4 protein expression was significantly upregulated in the lung tissue of SAP rats relative to the CON group, whereas QYD partly reversed these alterations (Supplementary File S6). Our data further demonstrated that QYD countered SAP-induced upregulation of Fe^2+^ (Figure 4(g)), MDA (Figure 4(h)), and 8-OHdG (Supplementary File S7) and downregulation of GSH (Figure 4(i)) in lung tissue, which are characteristic indicators of ferroptosis. In addition, rescue experiments confirm that Era eliminated the protective effect of QYD against pathological damage in lung tissue. Similarly, serum levels of TNF-*α* and IL-6 were elevated in SAP rats, and QYD could partly inhibit the production of inflammatory factors. Era, in contrast, prevented the downregulation of QYD (Supplementary File S8). These findings support the potential therapeutic effect of QYD on SAP-associated lung injury by enhancing ALDH2 activity to inhibit ferroptosis and apoptosis.

### 3.5. QYD Limits Neutrophil Infiltration by Increasing Active Form of AnxA1

**Figure 5:**
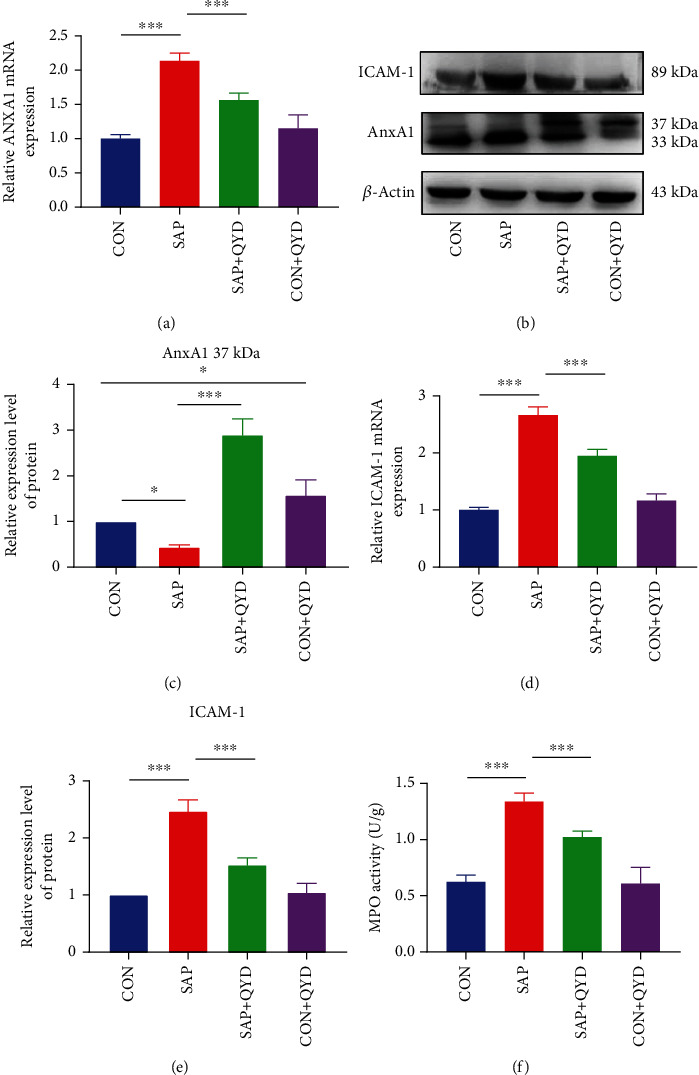
QYD decreases neutrophil infiltration via upregulating active AnxA1. (a) Analysis of the relative levels of AnxA1 mRNA in lung tissues using quantitative RT-PCR. (b, c) Western blotting analysis of the protein expression levels of AnxA1 in the lung tissues. (d, e) Analysis of the relative levels of ICAM-1 mRNA and protein in lung tissues. (f) MPO activity in the lung tissues. Data are presented as representative images or as the mean ± SD of each group of rats (*n* = 6 per group) from at least three separate experiments. ^∗^*P* < 0.05 and ^∗∗∗^*P* < 0.001.

Besides ALDH2, AnxA1 and ICAM-1 are also predicted core targets of QYD intervention in SAP-associated ALI. AnxA1 is a calcium-dependent phospholipid-binding protein, existing in two primary forms, a 33-kDa inactive form and a 37-kDa bioactive, which inhibits neutrophil tissue accumulation by reducing leukocyte infiltration and activating neutrophil apoptosis, with anti-inflammatory and tissue-protective properties. ICAM-1 is one of the essential leukocyte-endothelial cell adhesion molecules, and its elevated expression in ALI has become a marker of inflammatory cell migration and endothelial barrier injury. As shown in Figure 5(a), the mRNA expression of AnxA1 was elevated in the SAP group compared to the CON group and decreased after QYD treatment. Western blotting, as we expected, revealed that AnxA1 displayed two distinct bands. The expression of AnxA1 with a molecular weight of 37 kDa was reduced, whereas that of 33 kDa was raised in the SAP group compared to the CON group. After QYD gavage, we noticed an increase in intact AnxA1 (Figures 5(b) and 5(c)). Meanwhile, we found that ICAM-1 expression level was elevated in the SAP group, while QYD greatly reduced the mRNA and protein expression levels of ICAM-1 (Figures 5(d) and 5(e)). In addition, MPO activity, a marker of neutrophil infiltration, was elevated in the SAP rats, whereas QYD dramatically decreased MPO activity (Figure 5(f)). These findings imply that QYD may contribute to the inhibition of neutrophil infiltration and restoration of lung inflammation by posttranslationally regulating AnxA1 protein levels.

### 3.6. Effects of QYD on Lung Metabolism

**Figure 6:**
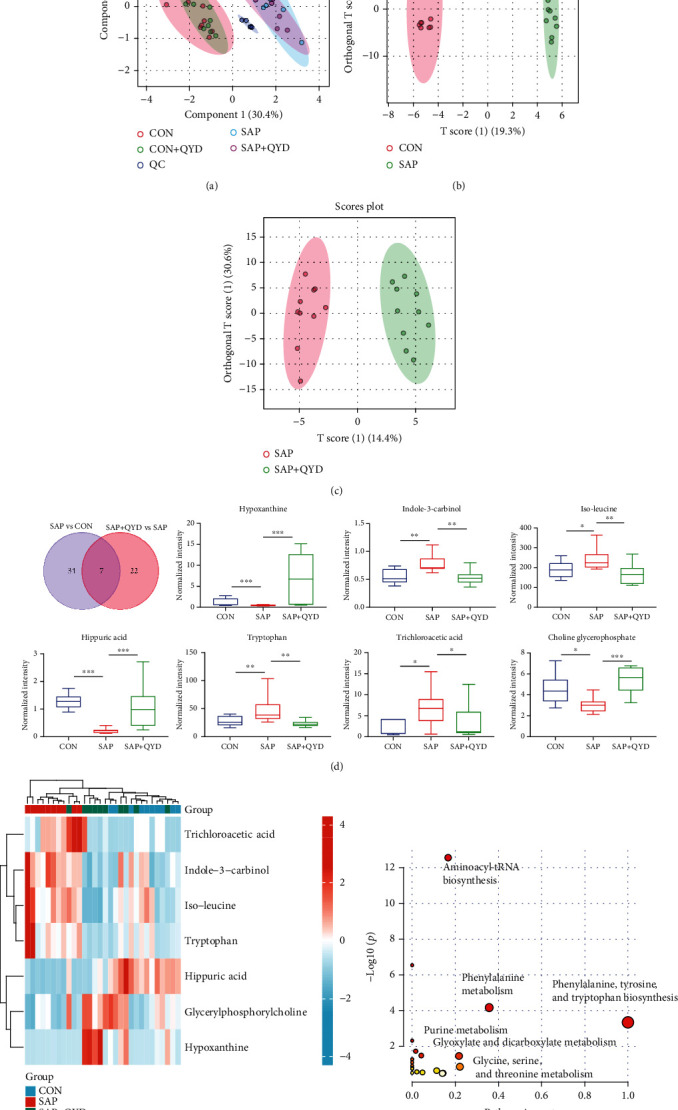
Effect of QYD on the lung metabolites of SAP rats. (a) PLS-DA score plots of the CON group, SAP group, SAP+QYD group, and CON+QYD group. (b) OPLS-DA score plots of the SAP vs. CON groups. (c) OPLS-DA score plots of the SAP+QYD vs. SAP groups. (d) Venn diagram of differential metabolites between SAP vs. CON groups and SAP+QYD vs. SAP groups. Effects of QYD on 7 differential metabolites levels in rats. (e) Heat map of 7 metabolites in each sample. (f) Analysis of metabolic pathway of differential metabolites between the QYD and SAP groups. Data are presented as the mean ± SD. *n* = 10 per group. ^∗^*P* < 0.05, ^∗∗^*P* < 0.01, and ^∗∗∗^*P* < 0.001.

PLS-DA is a supervised data analysis approach that overlooks intragroup and random errors in favor of focusing on sample variability between groups. The samples from the CON group were substantially separated from the SAP group in the PLS-DA model of this study, demonstrating the metabolic phenotype of lung tissue is significantly altered in STC-induced SAP rats. The QYD group was closer to the CON group, indicating that QYD may modulate those metabolites changed during SAP to return to normal levels. Furthermore, the overlap in the PLS-DA plot between the CON+QYD group and the CON group indicated that QYD had no significant effects on the metabolic phenotype of normal rats (Figure 6(a)).

The OPLS-DA method was adopted to investigate the effect of QYD on the regulation of metabolites in lung tissue. Differentiation between CON and SAP samples showed an altered metabolic profile of lung tissue in rats during STC-induced SAP (Figure 6(b)). Similarly, a significant trend of separation was also seen between SAP and QYD samples (Figure 6(c)). The VIP, FC, and *P* values were then used to screen the samples from each group for differential metabolites; the screening conditions are detailed in Materials and Methods. Compared to the CON group, SAP affected the relative intensities of 41 metabolites in the lungs (4 from the negative ion model and 37 from the positive ion model). In comparison to the SAP group, the QYD group changed 29 metabolites. Among these, seven intersecting differential metabolites were extracted by the Venn diagram, including choline glycerophosphate, hippuric acid, hypoxanthine, indole-3-carbinol, isoleucine, trichloroacetic acid, and tryptophan, and their changes in the relative intensities could be seen in Figure 6(d). Heat maps were used to visualize each group's relative abundance of differential metabolites (Figure 6(e)). The functional enrichment analysis results of the differential metabolites between the QYD and SAP groups indicated that potential pathways were related to aminoacyl-tRNA biosynthesis, phenylalanine metabolism, phenylalanine, tyrosine and tryptophan biosynthesis, purine metabolism, glyoxylate and dicarboxylate metabolism, and glycine, serine, and threonine metabolism (Figure 6(f)).

### 3.7. Effects of QYD on Lung Microbes

**Figure 7:**
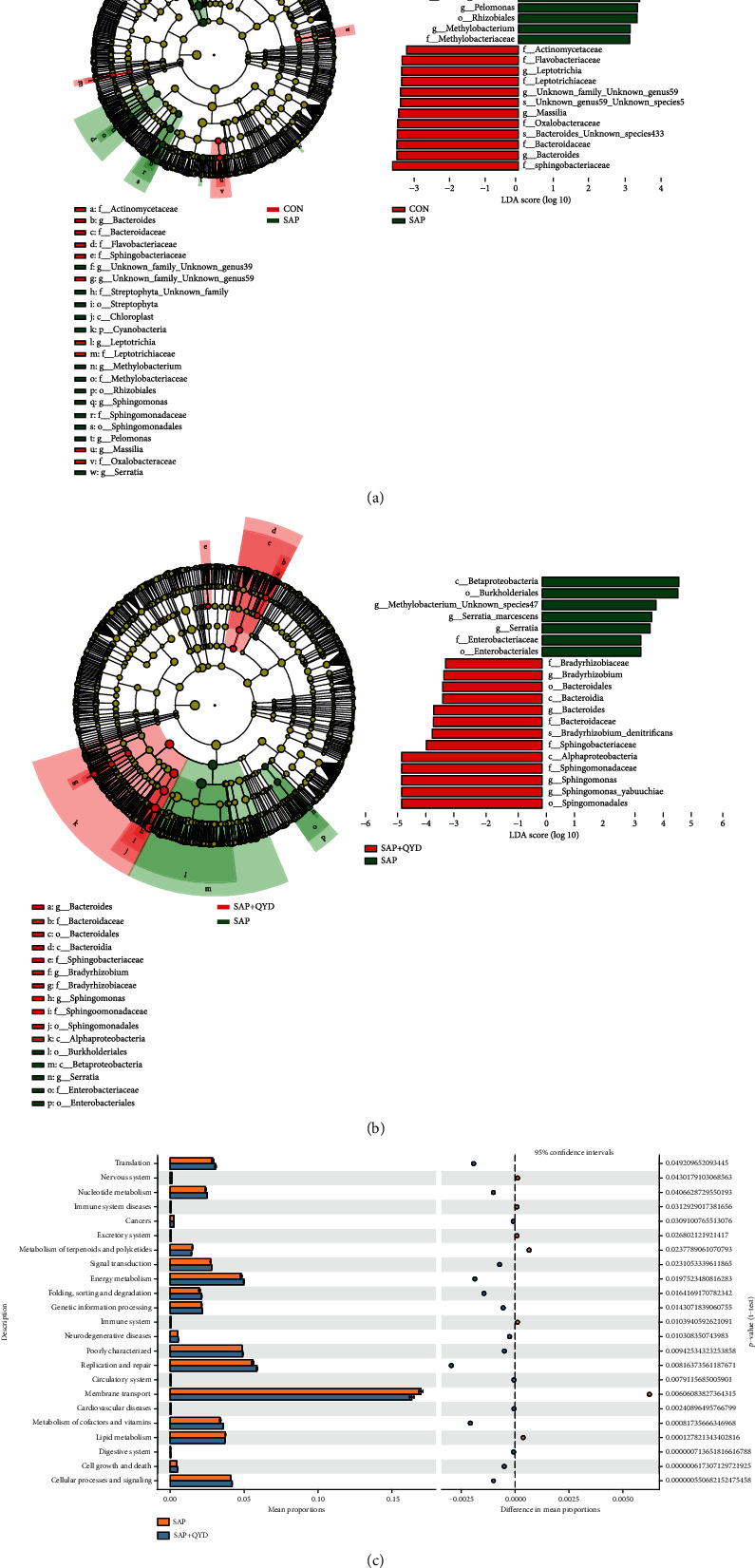
Effect of QYD on the lung microbiota composition of SAP rats. (a) Linear discriminant analysis between the SAP and CON groups (LDA score ≥ 3, *P* < 0.05). (b) Linear discriminant analysis between the QYD and SAP groups (LDA score ≥ 3, *P* < 0.05). (c) Metabolic pathways predicted by PICRUSt analysis that were statistically different between the QYD and SAP groups. Data are presented as the mean ± SD. *n* = 10 per group.

Alpha diversity was used to analyze the microbial diversity within the samples. Although there was no statistically significant difference between the SAP and CON groups, the data revealed that all three indices were reduced in the SAP group. When comparing the QYD group to the SAP group, the microbiome abundance did increase, albeit only a little. Please refer to Supplementary File S9. Differential microbe composition between the study groups was determined using LEfSe. On a phylum level, the relative abundance of Cyanobacteria increased in the SAP group than in the CON group. On a genus level, the SAP group had a higher relative abundance of Sphingomonas, Serratia, Pelomonas, and Methylobacterium and a lower relative abundance of Bacteroides than the CON group (Figure 7(a)). It is worth noting that in the QYD group, Bacteroides and Bradyrhizobium increased at the genus level compared to the SAP group (Figure 7(b)). The pulmonary microbiome's metabolic function was predicted using PICRUSt2. The pathways selected from the KEGG database were shown off. Significant differences were observed between the SAP and QYD groups in 23 level 2 pathways, including cell growth and death, cellular processes and signaling, digestive system, energy metabolism, excretory system, genetic information processing, immune system, and lipid metabolism (Figure 7(c)).

### 3.8. Integrative Analysis of the Ferroptosis, Metabolites, and Microbes

**Figure 8:**
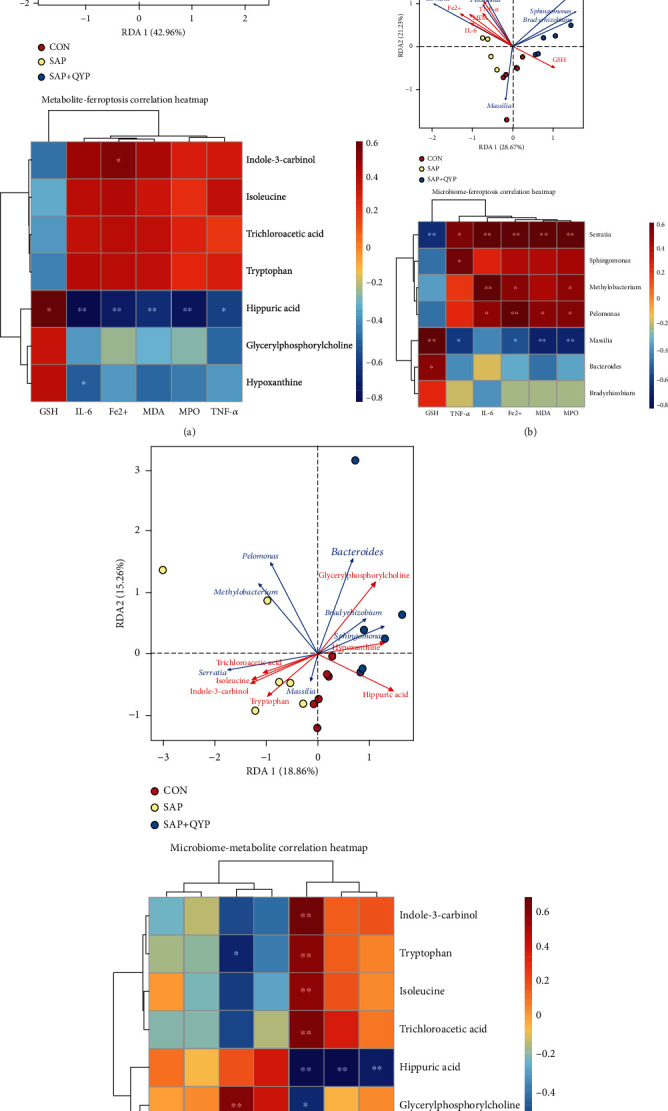
Correlation analysis of ferroptosis, metabolites, and microbiota. (a) Ferroptosis and metabolites. (b) Ferroptosis and microbiota. (c) Microbiota and metabolites. Each point in the RDA plot represents a sample, and the connecting arrows' length indicates the correlation's magnitude. The angle between the connecting arrows indicates the correlation, with acute angles indicating positive correlations and obtuse angles indicating negative correlations. The smaller the angle, the higher the correlation. The red color in the heat map indicates a positive correlation, while the blue indicates a negative one. ^∗^*P* < 0.05 and ^∗∗^*P* < 0.01.

Next, a complete investigation was conducted to determine the link between ferroptosis indicators, differential metabolites, and differential bacterial genera in rats. First, the connection between differential metabolites and ferroptosis markers was investigated. Specifically, hippuric acid was negatively connected with Fe^2+^, MDA, and inflammatory markers (IL-6, TNF-*α*, and MPO) and positively correlated with GSH levels. The concentration of Fe^2+^ was positively associated with that of indole-3-carbinol. See Figure 8(a). Also, we linked ferroptosis markers to differential bacterial genera (Figure 8(b)). The following combinations of ferroptosis and bacterial genera showed a significant correlation: Serratia-Fe^2+^ (positive), Serratia-MDA (positive), Serratia-GSH (negative), Pelomonas-Fe^2+^ (positive), Pelomonas-MDA (positive), Methylobacterium-Fe^2+^ (positive), Massilia-Fe^2+^ (negative), Massilia-MDA (negative), and Massilia-GSH (positive). Figure 8(b) also depicts the results of an investigation of the relationships between the bacterial genera and inflammatory markers. Lastly, we linked differential metabolites in lung tissue with bacterial genera. Methylobacterium-hippuric acid was the metabolite-microbe combination with the strongest connection among those studied (Figure 8(c)).

## 4. Discussion

QYD for treating SAP patients has demonstrated distinct advantages in suppressing the inflammatory response, enhancing gastrointestinal function, lowering infection rates, and facilitating quick recovery [24–26]. Several basic investigations have shown the molecular mechanisms of QYD in the treatment of SAP and associated distant organ damage. Wei et al.'s network pharmacology study demonstrated that QYD might attenuate pancreatic injury during SAP by inhibiting NF-*κ*B-mediated inflammatory response [10]. By suppressing intestinal sPLA2 expression, QYD dramatically reduced AP-induced intestinal barrier damage [27]. Furthermore, QYD prevented SAP-induced myocardial injury [28]. However, there have been fewer investigations on QYD's effectiveness against SAP-associated ALI. In our work, 5% of STC injected retrogradely into the biliopancreatic duct resulted in severe pancreatic and pulmonary histopathological injury. This method of establishing a rat model of SAP-associated ALI is stable and repeatable. QYD effectively ameliorated pancreatic and lung tissue injury in SAP, inhibited the release of inflammatory cytokines, alleviated pulmonary edema, and restored respiratory function.

SAP-associated ALI is an indirect secondary lung injury, the mechanism of which remains incompletely understood. It has been proposed that the pulmonary air-blood barrier is disrupted by inflammatory mediators from the circulation [2]. Many activated immune cells enter the lung and produce excess inflammatory factors, ROS, and damage-associated molecular patterns, causing damage and death of lung parenchymal cells. In our study, ALDH2, AnxA1, and ICAM-1 were identified to play an important role in the pathogenesis of lung injury followed by SAP and are potential core targets of QYD against SAP-associated ALI. This antagonistic mechanism may depend on the regulation of ferroptosis and neutrophil infiltration by QYD.

ALDH2 is a member of the acetaldehyde dehydrogenase family, and its gene polymorphisms are related to alcoholic pancreatitis susceptibility, particularly in Asian populations [29]. Previously, Cao et al. found that Alda-1 (an activator of ALDH2) decreased pancreatic enzyme, MDA, and 4-hydroxy nonanoic acid levels in mice with cerulein-induced mild AP and delayed apoptosis of acinar cells via the Bax/Bcl-2 pathway [30]. Many investigations have demonstrated that Alda-1 protects against hyperoxia- or heatstroke-induced ALI. Alda-1 has lung-protective effects by inhibiting ROS generation and endothelial inflammation in the blood vessels [31, 32]. Our work showed that mRNA and protein expressions of ALDH2 were considerably decreased in SAP-associated ALI rats' lung tissue. QYD enhanced the transcription and translation of the *aldh2* gene, which was consistent with the NP analysis. In addition, mitochondria-mediated apoptosis is intrinsically linked to the mitochondrial oxidoreductase ALDH2, which scavenges reactive aldehydes and reduces cellular oxidative damage. Multiple studies have indicated that increasing ALDH2 expression alleviates AP [30], myocardial infarction [33], atherosclerosis [34], heart failure [35], and ALI [32] by inhibiting apoptosis. Our study showed that QYD significantly reduced apoptosis in lung parenchymal cells caused by SAP, suggesting that QYD may alleviate lung damage by decreasing apoptosis via elevation of ALDH2 expression. Ferroptosis is a novel form of programmed cell death caused by the accumulation of iron-dependent lipid peroxides. The significance of ferroptosis in the development of SAP is crucial. The premature activation of trypsin is the first trigger for the onset of AP, and there is evidence that trypsin enhances the sensitivity of pancreatic acinar cells to ferroptosis, which significantly exacerbates pancreatic damage during AP [36]. Inhibiting ferroptosis reduced intestinal injury, bacterial translocation, and renal injury induced by AP [37, 38]. Ferroptosis of lung tissue, especially in alveolar epithelial cells, has been linked to ALI [39, 40]. Lung tissue GPX4 activity was dramatically suppressed in SAP-associated ALI, and ferroptosis aggravated lung damage [41]. Inhibiting the occurrence of ferroptosis is a potential strategy to combat SAP-associated ALI [42, 43]. Recently, ferroptosis has shown to be regulated by ALDH2. Researchers found that ALDH2 inhibits Alzheimer's disease-induced myocardial injury by regulating lipid peroxidation and ACSL4-dependent ferroptosis [44]. As a result, we performed downstream functional experiments. In rats with SAP-associated ALI, ferrous ions, 8-OHdG, MDA, and NCOA4 were expressed at higher levels, whereas GSH activity, SLC7A11, FTH1, and GPX4 proteins were expressed at lower levels. QYD therapy improved SAP-induced ferroptosis in lung tissues, as predicted. As shown by our rescue experiment (Supplementary File S8), Era counteracted the protective effect of QYD on lung damage and inflammatory response in SAP-associated ALI rats. In a word, our data shows that QYD may protect against SAP-induced pulmonary ferroptosis and apoptosis by promoting the ALDH2 expression.

The other two targets are linked to neutrophil infiltration. Neutrophils are widely recognized for being among the first immune cells to rush to the site of damage following the initiation of inflammation [45]. Similarly, one of the features of SAP-associated lung damage is significant neutrophil infiltration. Elastase, ROS, and neutrophil extracellular traps are the main culprits in SAP-associated SIRS [46, 47]. AnxA1 is a glucocorticoid downstream mediator with anti-inflammatory and tissue-healing properties. AnxA1 and its mimetic peptide (Ac2-26) inhibit NF-*κ*B and eNOS signaling to minimize neutrophil infiltration and improve oxidative damage in lung tissue [48]. Our findings show that SAP increased AnxA1 mRNA expression in rat lung tissues, indicating that a robust inflammatory response encouraged *anxa1* transcription. AnxA1 protein, which has anti-inflammatory action, did not show increased expression after the occurrence of SAP. During severe inflammation, elastase released from neutrophils promotes the cleavage of the 37 kDa fragment form of AnxA1, which has anti-inflammatory activity, to the 33 kDa fragment, which has no anti-inflammatory action [49]. Similarly, upon rapid migration of neutrophils to injury sites after SAP induction, intact AnxA1 (37 kDa) is cleaved by elastase and proteinase-3 to the inactive form (33 kDa), losing its inhibitory effect on neutrophil recruitment and leading to a stormy accumulation of neutrophils in lung tissues. Following QYD treatment, when the number of neutrophils infiltrating the lung is gradually reduced, the 37 kDa form of AnxA1 with anti-inflammatory activity rises, further limiting neutrophil recruitment and the production of proinflammatory mediators and promoting the subsidence of inflammation. This suggests that QYD blocks the neutrophil-amplified inflammatory cascade by inhibiting the cleavage of active AnxA1. ICAM-1 is a key adhesion molecule that recruits neutrophils, and its expression is one of the markers of neutrophil infiltration. Knockdown of ICAM-1 inhibits the development of ALI [50]. ICAM-1 expression was increased in the SAP-associated ALI model, whereas QYD therapy decreased ICAM-1 expression. In conclusion, our findings show that QYD may have lung-protective effects by increasing AnxA1 expression, inhibiting neutrophil infiltration and excessive inflammatory response.

Metabolomics is one of the applied approaches used for describing, at the metabolite level, the molecular processes of QYD against SAP-associated ALI. Hippuric acid is a significantly differentially expressed metabolite in our study. Hippuric acid is converted from phenolic compounds in the diet and is a normal component of urine. The relationship between hippuric acid and energy metabolism is tight. Any factor that affects mitochondrial oxidative phosphorylation may impact hippuric acid production [51]. Compared with the CON group, the expression of hippuric acid was downregulated in lung tissue during SAP, which may be associated with impaired energy metabolism in the lung during ALI. QYD upregulated the expression of hippuric acid, suggesting its ability to counteract mitochondrial dysfunction. In the correlation analysis, hippuric acid was negatively correlated with Fe^2+^ and MDA and positively correlated with GSH levels. This finding further suggests that improving energy metabolism and counteracting ferroptosis may be one of the mechanisms by which QYD intervenes SAP and improves ALI. In addition, this study found that aminoacyl-tRNA biosynthesis, phenylalanine metabolism, phenylalanine, tyrosine and tryptophan biosynthesis, purine metabolism, glyoxylate and dicarboxylate metabolism, and glycine, serine, and threonine metabolism are the primary metabolic pathways involved in QYD to improve SAP-associated ALI. The clearance of acetaldehyde and reactive oxygen species is tightly connected with glyoxylate and dicarboxylic acid metabolism. Intriguingly, our NP analysis revealed that QYD regulates critical targets, including ALDH2, in rats with SAP-associated ALI. In addition, two recent studies revealed that the glyoxylate and dicarboxylic acid metabolism are significantly altered in the lung tissue of mice with fine particle-associated lung injury and the serum of rats with radiation-associated lung injury [52, 53]. Tryptophan is an essential amino acid consumed only through the diet and produces active metabolites via the kynurenine or serotonin pathway. Tryptophan expression was significantly elevated in SAP-associated ALI, consistent with previous reports showing that kynurenine pathways are significantly increased during the AP-induced SIRS. Inhibition of kynurenine prevents extrapancreatic organ damage [54]. These findings confirm that QYD exerts anti-inflammatory effects by affecting the metabolic phenotype and ferroptosis.

Numerous studies demonstrate that the lung microbiome influences the development of illness by regulating immune responses. Before this study, Kyo et al. discovered a substantial decrease in alpha diversity of lung microbiome in the bronchoalveolar lavage fluid (BALF) from ARDS patients [55]. This parallels our results. SAP partially influenced the structure of the lung microbiome. After QYD intervention, the abundance of the lung microbiome partially increased. Kyo et al. identified a distinct microbial community structure represented by Betaproteobacteria, linked with elevated serum IL-6 levels and hospital mortality in patients with ARDS [55]. Our analysis found strong correlations between Sphingomonas, Serratia, Pelomonas, Methylobacterium, and Massilia and serum inflammatory markers (TNF-*α*, IL-6, and MPO) in rats. The difference in the bacterial genus may be associated with the variation in samples (BALF vs. lung tissue). Interestingly, we also discovered relationships between bacterial genera and ferroptosis in rats. Simultaneously, the connection between Methylobacterium and differential metabolites was discovered. Clearly, further research is required to understand the exact processes. The investigation of the lung microbiome composition at the phylum level indicated that the abundance of Firmicutes was dramatically reduced in the SAP group relative to the CON group. The Firmicutes maintain the homeostasis of the immune system, and their lower abundance was directly linked to inflammatory reactions. The degree of dysbiosis was closely related to the abundance of Firmicutes [56]. The increased abundance of Firmicutes in the QYD group compared to the SAP group indicated that dysbiosis was obtained. Finally, we conclude that QYD reduces SAP-induced lung injury by partly influencing the lung microbiome.

This research performed a multidimensional analysis to parse the potential mechanism of QYD against SAP-associated ALI from the perspective of systems biology, but there are still several limitations. First, we hypothesized that QYD exerts a pulmonary protective effect via promoting the ALDH2 expression and, by extension, suppressing ferroptosis. However, it is not yet known whether inhibition of ALDH2 expression can reverse the inhibitory effect of QYD on ferroptosis. The mechanism by which ALDH2 regulates signaling molecules associated with ferroptosis requires additional exploration. Inhibiting neutrophil infiltration and NET formation, QYD has anti-inflammatory actions. We solely investigated MPO activity in lung tissue. Indeed, the inhibitory effect of QYD on NET formation should be determined in the future as soon as feasible. Secondly, in the study of metabolites and microorganisms, we identified some differential metabolites and microbes associated with ferroptosis and inflammation. However, the substantial link between differential metabolites and microbes and ferroptosis remains poorly characterized. Further research is required to determine if QYD suppresses inflammatory responses by modulating microbe-metabolite-ferroptosis, such as applying metabolites and microbes during SAP therapy. Furthermore, the molecular mechanisms behind the involvement of QYD-related active components, such as palbinone and pulmatin, in SAP rats and the regulation of ferroptosis remain unknown. Our future endeavors will center on this.

## 5. Conclusion

In conclusion, network pharmacology, metabolomics, and 16s rDNA sequencing were used to evaluate the molecular mechanisms of QYD against SAP-associated ALI. Functional enrichment and correlation analysis revealed the importance of ferroptosis, metabolic, and microbial function in QYD's lung protective effects. This integrated analysis method is suitable for the study of TCM and offers a fresh paradigm for future research on the therapeutic mechanisms of TCM.

## Data Availability

The data used to support the findings of this study are available from the corresponding authors.

## Abbreviations

AP: Acute pancreatitis
SAP: Severe acute pancreatitis
SIRS: Systemic inflammatory response syndrome
MODS: Multiple organ dysfunction syndromes
ALI: Acute lung injury
ARDS: Acute respiratory distress syndrome
QYD: Qingyi decoction
NP: Network pharmacology
GSH: Glutathione
MDA: Malondialdehyde
MPO: Myeloperoxidase
Era: Erastin
TUNEL: TdT-mediated dUTP nick end labeling
DEPs: Differential expression proteins
DEGs: Differentially expressed genes
ALDH2: Acetaldehyde dehydrogenase 2
AnxA1: Annexin A1
FTH1: Ferritin heavy chain 1
NCOA4: Nuclear receptor coactivator 4
SLC7A11: Solute carrier family 7 member 11
8-OHdG: 8-Hydroxy-2′-deoxyguanosine
ICAM-1: Intercellular cell adhesion molecule-1
STC: Sodium taurocholate
ROS: Reactive oxygen species
GPX4: Glutathione peroxidase 4
NET: Neutrophil extracellular trap
TCM: Traditional Chinese medicine.

## Supplementary Materials

Supplementary Materials Supplementary File S1: 225 ingredients and 514 potential targets for QYD. Supplementary File S2: the sequences of the primers for qRT-PCR. Supplementary File S3: details of the analytical conditions and data preprocessing for mass spectrum. Supplementary File S4: the CDOCKER interaction energy of all ingredients and proteins. Supplementary File S5: apoptosis in the lung tissue of each group of rats. Supplementary File S6: expression of ferroptosis-related proteins in lung tissue of rats in each group. Supplementary File S7: expression of 8-OHdG in lung tissue of rats in each group. Supplementary File S8: effects of QYD and/or erastin on lung tissue damage and inflammation in SAP rats. Supplementary File S9: effect of QYD on the Shannon index, Simpson index, and Chao1 index of SAP rats. Graphical abstract: protective mechanism of QYD in SAP-associated ALI rat model.
