# Supplementary material for: Ferroptosis in Rat Lung Tissue during Severe Acute Pancreatitis-Associated Acute Lung Injury: Protection of Qingyi Decoction
Source: Oxid Med Cell Longev. 2023 Feb 11;2023:5827613. doi: 10.1155/2023/5827613 (PMC9938780; doi:10.1155/2023/5827613)
Supplement: Supplementary Materials — Supplementary File S1: 225 ingredients and 514 potential targets for QYD. Supplementary File S2: the sequences of the primers for qRT-PCR. Supplementary File S3: details of the analytical conditions and data preprocessing for mass spectrum. Supplementary File S4: the CDOCKER interaction energy of all ingredients and proteins. Supplementary File S5: apoptosis in the lung tissue of each group of rats. Supplementary File S6: expression of ferroptosis-related proteins in lung tissue of rats in each group. Supplementary File S7: expression of 8-OHdG in lung tissue of rats in each group. Supplementary File S8: effects of QYD and/or erastin on lung tissue damage and inflammation in SAP rats. Supplementary File S9: effect of QYD on the Shannon index, Simpson index, and Chao1 index of SAP rats. Graphical abstract: protective mechanism of QYD in SAP-associated ALI rat model. [file 5827613.f1.zip › Supplementary File S1.docx]

| 202 DEGs | 514 QYD-Targets |
| --- | --- |
| HIST2H3C2 | AADACL2 |
| HMGA1 | ABAT |
| LOC100911295 | ABCB1 |
| HSPB6 | ABCG2 |
| FKBP10 | ABL1 |
| RGD1560065 | ABO |
| SFTPA1 | ACACA |
| S100A1 | ACADSB |
| RPL36 | ACHE |
| YBX1-PS3 | ACOX1 |
| H1-5 | ACPP |
| ATP5IF1 | ACSL3 |
| AHSP | ACSL4 |
| LOC100361913 | ACTB |
| OTUD6B | ADH1C |
| H3F3C | ADH5 |
| RPS24 | ADRA1A |
| RRBP1 | ADRA1B |
| S100G | ADRA1D |
| S100A4 | ADRA2A |
| SERPINA6 | ADRA2B |
| DIXDC1 | ADRA2C |
| TBCA | ADRB1 |
| CHMP4BL1 | ADRB2 |
| CLTA | AHR |
| SFTPD | AHSA1 |
| RCG_63270 | AKR1B1 |
| H4C2 | AKR1C1 |
| H4F16 | AKR1C2 |
| HIST1H4M | AKR1C3 |
| NMT2 | AKR1D1 |
| SRSF4 | AKT1 |
| SUMO3 | ALB |
| CREM | ALDH1A1 |
| SERBP1 | ALDH1A2 |
| MB | ALDH2 |
| NCL | ALDH3A1 |
| DNAJC8 | ALDH5A1 |
| FAAH | ALOX12 |
| RPS25 | ALOX5 |
| HSPE1 | ANXA1 |
| RPS21 | APOD |
| NPM1 | AR |
| SRSF3 | ARF1 |
| EIF4B | ARF6 |
| PDCD5 | ATP2A1 |
| TAF15 | ATP5A1 |
| MARCKS | ATP5B |
| ABCF1 | ATP5C1 |
| CETN3 | AURKB |
| PPP1R14A | BACE |
| GAP43 | BAX |
| RGD1311703 | BBC3 |
| ANP32A | BCHE |
| CNPY2 | BCL2 |
| TXN | BCL2L1 |
| SRSF1 | BIRC5 |
| NUCB2 | BMPR2 |
| PIGR | C8G |
| FBLIM1 | CA1 |
| CNBP | CA12 |
| ARPIN | CA14 |
| RPS3A | CA2 |
| JPT2 | CA3 |
| CDV3 | CA4 |
| APOBR | CA5A |
| MAP7D1 | CA5B |
| CHCHD4 | CA6 |
| CRK | CA7 |
| HSBP1 | CA9 |
| HMGB3 | CACNA1S |
| TCOF1 | CACNA2D1 |
| ZC3H18 | CALM |
| SFRS7 | CALM1 |
| CLIP1 | CALY |
| FUBP1 | CASP3 |
| JCAD | CASP8 |
| TRA2B | CASP9 |
| S100A6 | CAT |
| LRRFIP1 | CAV1 |
| CDKN2C | CBR1 |
| PALMD | CCL2 |
| TMOD1 | CCNA2 |
| RPL3 | CCNB1 |
| VEGF | CCND1 |
| CD2BP2 | CD40LG |
| NSFL1C | CDK1 |
| RBPMS | CDK2 |
| RBM8A | CDK6 |
| HMGN5 | CDK7 |
| EEF1B2 | CDKN1A |
| LSM3 | CEBPB |
| FKBP7 | CES1 |
| ALDH2 | CHEK1 |
| CALR | CHEK2 |
| RGD1304567 | CHRM1 |
| CHMP1A | CHRM2 |
| RCN2 | CHRM3 |
| UBE2R2 | CHRM4 |
| PEX19 | CHRM5 |
| PFDN1 | CHRNA2 |
| PEA15 | CHRNA3 |
| RGD1559962 | CHRNA4 |
| HDGFL3 | CHRNA7 |
| EPB41L2 | CHRNB2 |
| ARGLU1 | CHRNB4 |
| PSMD4 | CHUK |
| SH3BGRL | CLDN4 |
| ENY2 | CLEC4E |
| STMN1 | CNR1 |
| RPL13A | CNR2 |
| PURA | COL1A1 |
| PTMS | COL3A1 |
| NASP | COMT |
| DR1 | COX4I1 |
| ATG3 | COX5A |
| GLO1 | COX5B |
| SLTM | COX6A2 |
| SRSF2 | COX6B1 |
| SRSF6 | COX6C |
| UBE2N | COX7A1 |
| MAGED2 | COX7B |
| LOC103690005 | COX7C |
| SWI5 | COX8A |
| RILPL1 | CRH |
| ZNRD2 | CRP |
| NOLC1 | CRYZ |
| LOC100910678 | CSNK2A1 |
| RPL4 | CSNK2B |
| ZC2HC1A | CTRB1 |
| GIT2 | CTSB |
| AKAP2 | CTSD |
| RPL23 | CXCL10 |
| CTTN | CXCL11 |
| NUCKS1 | CXCL2 |
| PHAX | CXCL8 |
| TIMM8A1 | CYBA |
| RNF113A2 | CYBB |
| TRIOBP | CYCS |
| TNXB | CYP17A1 |
| MAP6 | CYP19A1 |
| RE1 | CYP1A1 |
| ZYX | CYP1A2 |
| MAPT | CYP1B1 |
| ABHD14B | CYP26A1 |
| MDH2 | CYP26B1 |
| ACTR2 | CYP26C1 |
| CAPZA1 | CYP27B1 |
| C9 | CYP2C8 |
| SIRPA | CYP2C9 |
| GSN | CYP2D6 |
| MTURN | CYP2E1 |
| PARVA | CYP3A4 |
| PPP2R5A | CYP3A43 |
| ICAM1 | CYP3A5 |
| RALB | CYP3A7 |
| DDAH2 | DAO |
| ALCAM | DCAF5 |
| ANXA2 | DHFRL1 |
| ANXA4 | DIO1 |
| ILF2 | DNMT1 |
| CHCHD3 | DPP4 |
| MUC5B | DRD1 |
| PKM | DRD2 |
| PLG | DRD3 |
| FGB | DRD4 |
| DPYSL2 | DRD5 |
| APOE | DUOX2 |
| RAB1A | E2F1 |
| GPX3 | E2F2 |
| COL12A1 | ECI2 |
| RT1-BB | EFTUD1 |
| IGH-1A | EGF |
| ITGB2 | EGFR |
| NMES1 | EGLN1 |
| KPNA4 | EIF3F |
| ASAH1 | EIF6 |
| LOC100363782 | ELK1 |
| GPX1 | ELOVL4 |
| CLIC3 | ERBB2 |
| INMT | ERBB3 |
| RAP1A | ESR1 |
| CAPG | ESR2 |
| DNASE2 | ESRRA |
| CAPZA2 | ESRRB |
| CTSC | ESRRG |
| KRT10 | F10 |
| PGAM1 | F3 |
| FTH1 | F7 |
| RAP2B | FABP5 |
| ANXA1 | FABP6 |
| CTSZ | FADS1 |
| KRT6A | FADS2 |
| FTL1 | FAH |
| CKMT1 | FASLG |
| SERPINA3N | FASN |
| BPIFB1 | fccA |
| STFA2 | FECH |
| CTSS | FFAR1 |
| ACTG2 | FKBP1A |
| CLCA1 | FN1 |
| A2M | FOS |
|  | FOSL1 |
|  | FOSL2 |
|  | FURIN |
|  | GABBR1 |
|  | GABRA1 |
|  | GABRA2 |
|  | GABRA3 |
|  | GABRA4 |
|  | GABRA5 |
|  | GABRA6 |
|  | GABRB1 |
|  | GABRB2 |
|  | GABRB3 |
|  | GABRD |
|  | GABRE |
|  | GABRG1 |
|  | GABRG2 |
|  | GABRG3 |
|  | GABRP |
|  | GABRQ |
|  | GJA1 |
|  | GJB1 |
|  | GLTP |
|  | GM2A |
|  | GNRH1 |
|  | GNRHR |
|  | GOT1 |
|  | GPBAR1 |
|  | GPER1 |
|  | GPRC5A |
|  | GRIA2 |
|  | GRIN1 |
|  | GRIN2A |
|  | GRIN2B |
|  | GRIN2C |
|  | GRIN2D |
|  | GRIN3A |
|  | GRIN3B |
|  | GRM1 |
|  | GRM5 |
|  | GSK3B |
|  | GSTA1 |
|  | GSTM1 |
|  | GSTM2 |
|  | GSTP1 |
|  | GUCA1A |
|  | HA |
|  | HAO1 |
|  | HAS2 |
|  | HCK |
|  | HDAC2 |
|  | HDAC9 |
|  | HIBCH |
|  | HIF1A |
|  | HK2 |
|  | HMGCR |
|  | HMOX1 |
|  | HNF4A |
|  | HNF4G |
|  | HOXA10 |
|  | HPGDS |
|  | HRSP12 |
|  | HSD11B1 |
|  | HSD11B2 |
|  | HSD17B1 |
|  | HSD17B11 |
|  | HSD3B1 |
|  | HSF1 |
|  | HSP90 |
|  | HSP90AA1 |
|  | HSPA2 |
|  | HSPA5 |
|  | HSPB1 |
|  | HTR1A |
|  | HTR1B |
|  | HTR1D |
|  | HTR2A |
|  | HTR2B |
|  | HTR2C |
|  | HTR3A |
|  | ICAM1 |
|  | IFNG |
|  | IGF2 |
|  | IGFBP3 |
|  | IGHG1 |
|  | IGHG2 |
|  | IKBKB |
|  | IL10 |
|  | IL1A |
|  | IL1B |
|  | IL2 |
|  | IL6 |
|  | INS |
|  | INSR |
|  | IRF1 |
|  | JAK1 |
|  | JUN |
|  | KANSL3 |
|  | KCNH2 |
|  | KCNMA1 |
|  | KDR |
|  | KLF7 |
|  | KRT12 |
|  | KRT2 |
|  | LALBA |
|  | LCN1 |
|  | LIP3 |
|  | LPL |
|  | LSS |
|  | LTA4H |
|  | LTB4R |
|  | LTB4R2 |
|  | LTF |
|  | LY96 |
|  | MAOA |
|  | MAOB |
|  | MAP2 |
|  | MAPK1 |
|  | MAPK10 |
|  | MAPK14 |
|  | MAPK8 |
|  | MAPK8IP1 |
|  | MCL1 |
|  | ME2 |
|  | MED1 |
|  | MGAM |
|  | MMP1 |
|  | MMP2 |
|  | MMP3 |
|  | MMP9 |
|  | MPO |
|  | MT-CO1 |
|  | MT-CO2 |
|  | MT-CO3 |
|  | MTTP |
|  | MYC |
|  | NCF1 |
|  | NCF2 |
|  | NCF4 |
|  | NCOA1 |
|  | NCOA2 |
|  | NFATC1 |
|  | NFE2L2 |
|  | NFKB1 |
|  | NFKB2 |
|  | NFKBIA |
|  | NKX3-1 |
|  | NOS2 |
|  | NOS3 |
|  | NOX5 |
|  | NPEPPS |
|  | NPPB |
|  | NPRS |
|  | NQO1 |
|  | NQO2 |
|  | NR0B1 |
|  | NR1H2 |
|  | NR1H3 |
|  | NR1H4 |
|  | NR1I2 |
|  | NR1I3 |
|  | NR3C1 |
|  | NR3C2 |
|  | OBP2A |
|  | ODC1 |
|  | OGDH |
|  | OLR1 |
|  | OPRD1 |
|  | OPRK1 |
|  | OPRM1 |
|  | ORM1 |
|  | PAEP |
|  | PARP1 |
|  | PCNA |
|  | PCOLCE |
|  | PDE10A |
|  | PDE3A |
|  | PDK4 |
|  | PGR |
|  | PGRMC1 |
|  | PIK3CG |
|  | PIM1 |
|  | PKIA |
|  | PLA2G1B |
|  | PLA2G2A |
|  | PLA2G2D |
|  | PLA2G2E |
|  | PLAT |
|  | PLAU |
|  | PMP2 |
|  | PON1 |
|  | POR |
|  | PPA_RS05235 |
|  | PPARA |
|  | PPARD |
|  | PPARG |
|  | PPP1CC |
|  | PPP3CA |
|  | PPP3R1 |
|  | PPT1 |
|  | PRDX5 |
|  | PRKACA |
|  | PRKCA |
|  | PRKCB |
|  | PRKCD |
|  | PRKCE |
|  | PRLR |
|  | PRSS1 |
|  | PSG5 |
|  | PSMD3 |
|  | PTEN |
|  | PTGER3 |
|  | PTGS1 |
|  | PTGS2 |
|  | PTK2B |
|  | PTPN1 |
|  | PVR |
|  | PYGM |
|  | RAB9A |
|  | RAC1 |
|  | RAC2 |
|  | RADH |
|  | RAF1 |
|  | RARA |
|  | RARB |
|  | RARG |
|  | RARRES1 |
|  | RASA1 |
|  | RASSF1 |
|  | RB1 |
|  | RBP4 |
|  | RCVRN |
|  | RELA |
|  | RHO |
|  | RORA |
|  | RUNX1T1 |
|  | RUNX2 |
|  | RUVBL2 |
|  | RXRA |
|  | RXRB |
|  | RXRG |
|  | S100B |
|  | SCN10A |
|  | SCN11A |
|  | SCN1A |
|  | SCN1B |
|  | SCN2A |
|  | SCN2B |
|  | SCN3A |
|  | SCN3B |
|  | SCN4A |
|  | SCN4B |
|  | SCN5A |
|  | SCN7A |
|  | SCN8A |
|  | SCN9A |
|  | SCO5081 |
|  | SEC14L2 |
|  | SELE |
|  | SERPINE1 |
|  | SF3B3 |
|  | SHBG |
|  | SIGMAR1 |
|  | SLC15A1 |
|  | SLC18A2 |
|  | SLC2A4 |
|  | SLC6A2 |
|  | SLC6A3 |
|  | SLC6A4 |
|  | SLC8A1 |
|  | SLCO1B3 |
|  | SLPI |
|  | SOAT1 |
|  | SOAT2 |
|  | SOD1 |
|  | SPP1 |
|  | SQLE |
|  | SREBF1 |
|  | STAT1 |
|  | STK17B |
|  | SULT1E1 |
|  | SULT2A1 |
|  | SULT2B1 |
|  | SYK |
|  | TAT |
|  | TDRD7 |
|  | TEP1 |
|  | TGFB1 |
|  | TH |
|  | THBD |
|  | TLR4 |
|  | TM1468 |
|  | TNF |
|  | TOP1 |
|  | TOP2 |
|  | TOP2A |
|  | TP53 |
|  | TPR |
|  | TRAPPC3 |
|  | TRPV1 |
|  | TRPV3 |
|  | TYR |
|  | UBA1 |
|  | UGT3A1 |
|  | VCAM1 |
|  | VCP |
|  | VDR |
|  | VEGFA |
|  | VKORC1 |
|  | VLDLR |
|  | XDH |
|  | YARS2 |
