# Supplementary material for: Ferroptosis in Rat Lung Tissue during Severe Acute Pancreatitis-Associated Acute Lung Injury: Protection of Qingyi Decoction
Source: Oxid Med Cell Longev. 2023 Feb 11;2023:5827613. doi: 10.1155/2023/5827613 (PMC9938780; doi:10.1155/2023/5827613)
Supplement: Supplementary Materials — Supplementary File S1: 225 ingredients and 514 potential targets for QYD. Supplementary File S2: the sequences of the primers for qRT-PCR. Supplementary File S3: details of the analytical conditions and data preprocessing for mass spectrum. Supplementary File S4: the CDOCKER interaction energy of all ingredients and proteins. Supplementary File S5: apoptosis in the lung tissue of each group of rats. Supplementary File S6: expression of ferroptosis-related proteins in lung tissue of rats in each group. Supplementary File S7: expression of 8-OHdG in lung tissue of rats in each group. Supplementary File S8: effects of QYD and/or erastin on lung tissue damage and inflammation in SAP rats. Supplementary File S9: effect of QYD on the Shannon index, Simpson index, and Chao1 index of SAP rats. Graphical abstract: protective mechanism of QYD in SAP-associated ALI rat model. [file 5827613.f1.zip › Supplementary File S3 (1).docx]

**Supplementary File S3.** Details of the analytical conditions and data pre-processing

The column temperature was 50 °C, and the flow rate was 0.35 mL/min. The mobile phases A and B were 0.1 % formic acid aqueous solution and 0.1 % formic acid acetonitrile solution, respectively. The elution gradient started from 10 % B and was held for 1 min, increasing linearly to 40 % B at 4 min and 100 % B at 15 min and was held for 4 min. The system was equilibrated for 4 min before the next injection. The injection volume was 5 μl. Lung tissue samples were separated by UPLC and analyzed by Q-Trap 5500 mass spectrometer. In the mass spectrometry system, CUR, GS1, and GS2 pressures were 0.241, 0.276, and 0.276 MPa, respectively, ESI+ and ESI- mode spray voltages were 4500 and 5500 V, respectively, and the scan m/z range was 70~1000. The MRM-Ion Pair Finder software tool based on the UPLC/Q-TOF MS was used for data conversion and software provided by the instrument vendor for data pre-processing, including peak identification, alignment, and matching.
