# Supplementary material for: Ferroptosis in Rat Lung Tissue during Severe Acute Pancreatitis-Associated Acute Lung Injury: Protection of Qingyi Decoction
Source: Oxid Med Cell Longev. 2023 Feb 11;2023:5827613. doi: 10.1155/2023/5827613 (PMC9938780; doi:10.1155/2023/5827613)
Supplement: Supplementary Materials — Supplementary File S1: 225 ingredients and 514 potential targets for QYD. Supplementary File S2: the sequences of the primers for qRT-PCR. Supplementary File S3: details of the analytical conditions and data preprocessing for mass spectrum. Supplementary File S4: the CDOCKER interaction energy of all ingredients and proteins. Supplementary File S5: apoptosis in the lung tissue of each group of rats. Supplementary File S6: expression of ferroptosis-related proteins in lung tissue of rats in each group. Supplementary File S7: expression of 8-OHdG in lung tissue of rats in each group. Supplementary File S8: effects of QYD and/or erastin on lung tissue damage and inflammation in SAP rats. Supplementary File S9: effect of QYD on the Shannon index, Simpson index, and Chao1 index of SAP rats. Graphical abstract: protective mechanism of QYD in SAP-associated ALI rat model. [file 5827613.f1.zip › Supplementary File S4 (1).docx]

**Supplementary File S4**. The CDOCKER interaction energy

| Ingredient | Target | -CDOCKER interaction energy (Kcal/mol) |
| --- | --- | --- |
| Chrysophanein | ALDH2 | 50.035 |
| 8-Methoxy-5-*O*-Glucoside Flavone | ALDH2 | 35.1106 |
| Pulmatin | ALDH2 | 41.1264 |
| Palbinone | AnxA1 | 42.2007 |
| Gardenolic Acid B | AnxA1 | 44.8839 |
| Quercetin | ICAM-1 | 45.9925 |
| Kaempferol | ICAM-1 | 43.4328 |
