# Supplementary material for: Ferroptosis in Rat Lung Tissue during Severe Acute Pancreatitis-Associated Acute Lung Injury: Protection of Qingyi Decoction
Source: Oxid Med Cell Longev. 2023 Feb 11;2023:5827613. doi: 10.1155/2023/5827613 (PMC9938780; doi:10.1155/2023/5827613)
Supplement: Supplementary Materials — Supplementary File S1: 225 ingredients and 514 potential targets for QYD. Supplementary File S2: the sequences of the primers for qRT-PCR. Supplementary File S3: details of the analytical conditions and data preprocessing for mass spectrum. Supplementary File S4: the CDOCKER interaction energy of all ingredients and proteins. Supplementary File S5: apoptosis in the lung tissue of each group of rats. Supplementary File S6: expression of ferroptosis-related proteins in lung tissue of rats in each group. Supplementary File S7: expression of 8-OHdG in lung tissue of rats in each group. Supplementary File S8: effects of QYD and/or erastin on lung tissue damage and inflammation in SAP rats. Supplementary File S9: effect of QYD on the Shannon index, Simpson index, and Chao1 index of SAP rats. Graphical abstract: protective mechanism of QYD in SAP-associated ALI rat model. [file 5827613.f1.zip › Supplementary File S6 (1).docx]

**
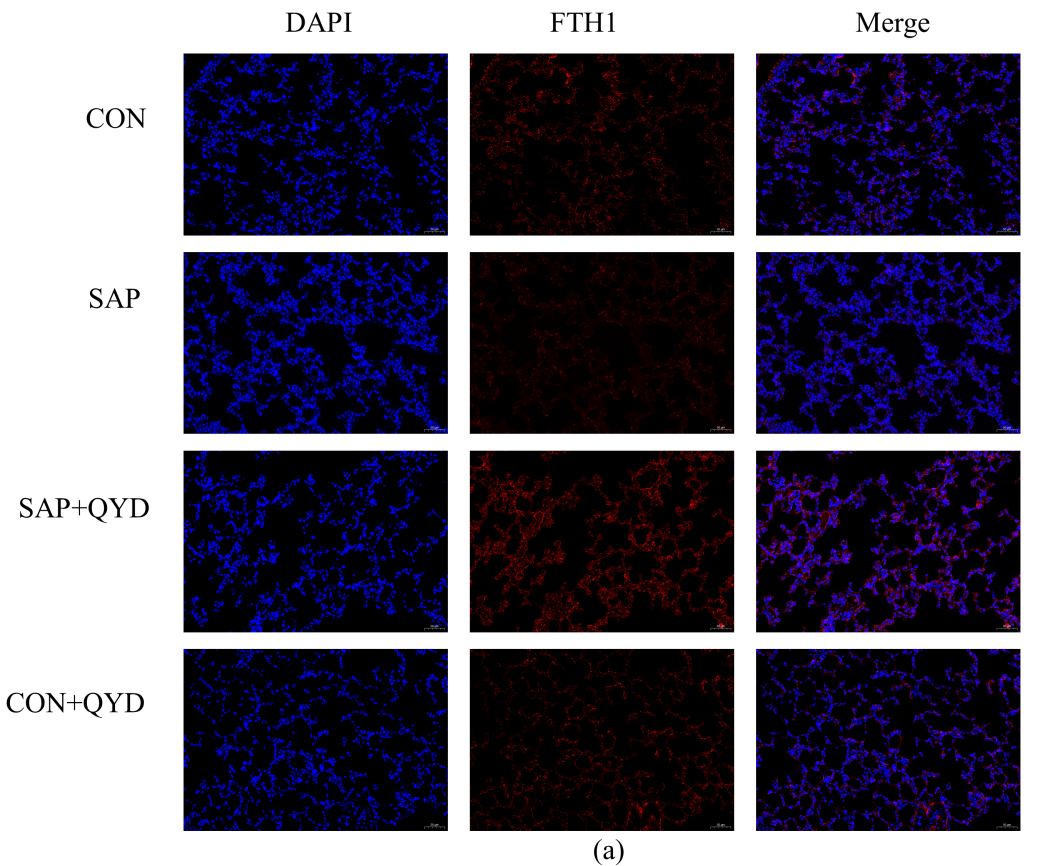
**

**
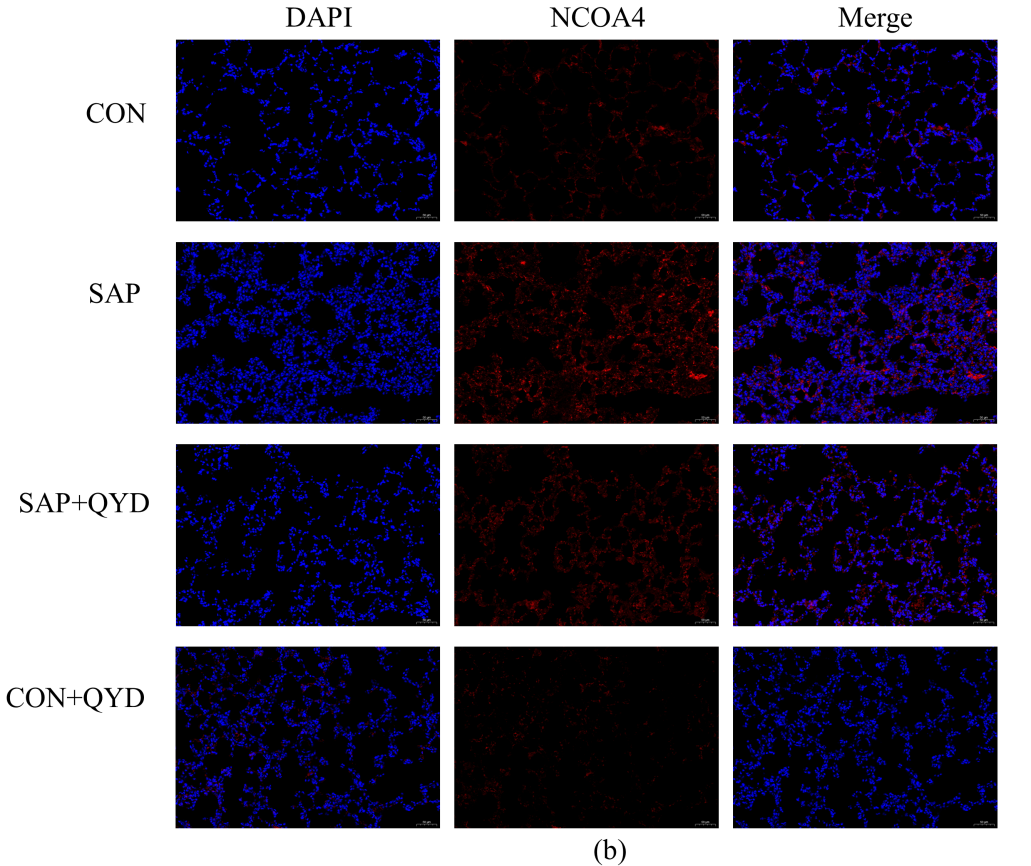
**

**
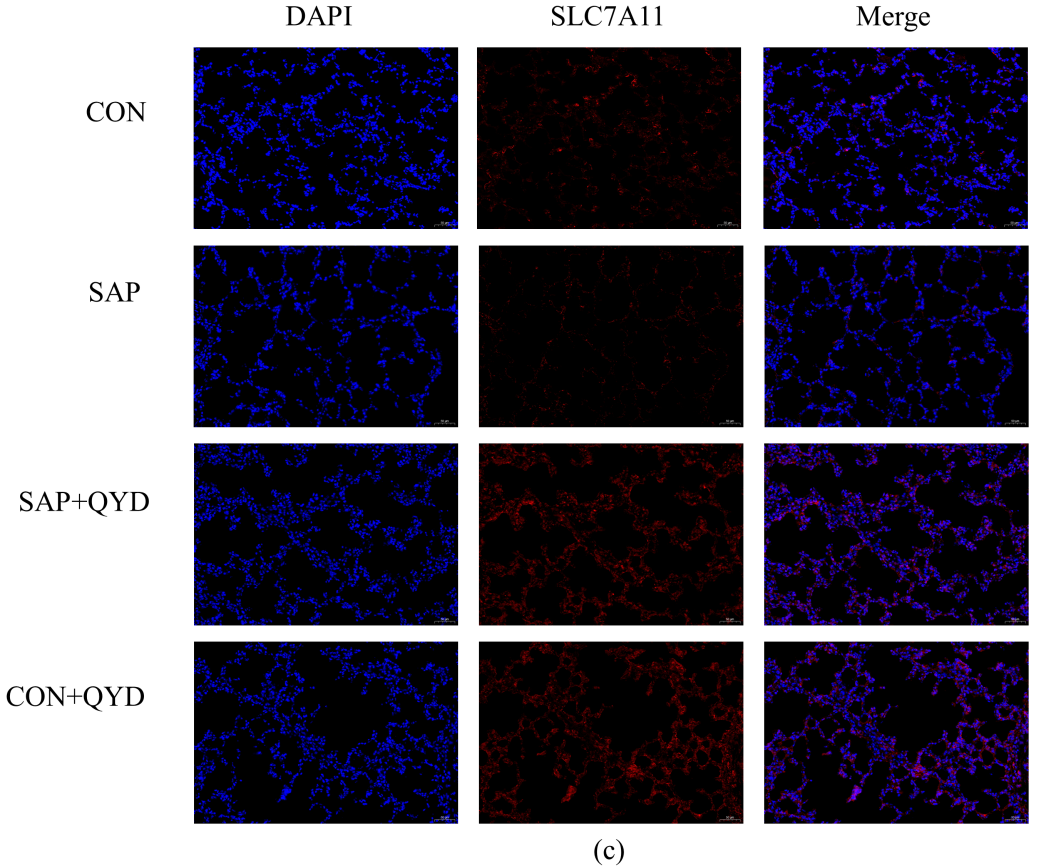
**

**Supplementary File S6**. Expression of ferroptosis-related proteins in lung tissue of rats in each group. (a) FTH1, DAPI, Merged images (scale bar, 50 μm). (b) NCOA4, DAPI, Merged images (scale bar, 50 μm). (c) SLC7A11, DAPI, Merged images (scale bar, 50 μm). Data are presented as representative images of each group of rats (*n* = 6 per group) from at least three separate experiments.
