# Supplementary figures and images for: Ferroptosis in Rat Lung Tissue during Severe Acute Pancreatitis-Associated Acute Lung Injury: Protection of Qingyi Decoction
Source: Oxid Med Cell Longev. 2023 Feb 11;2023:5827613. doi: 10.1155/2023/5827613 (PMC9938780; doi:10.1155/2023/5827613)

# Protective mechanism of QYD in SAP-associated ALI rat model

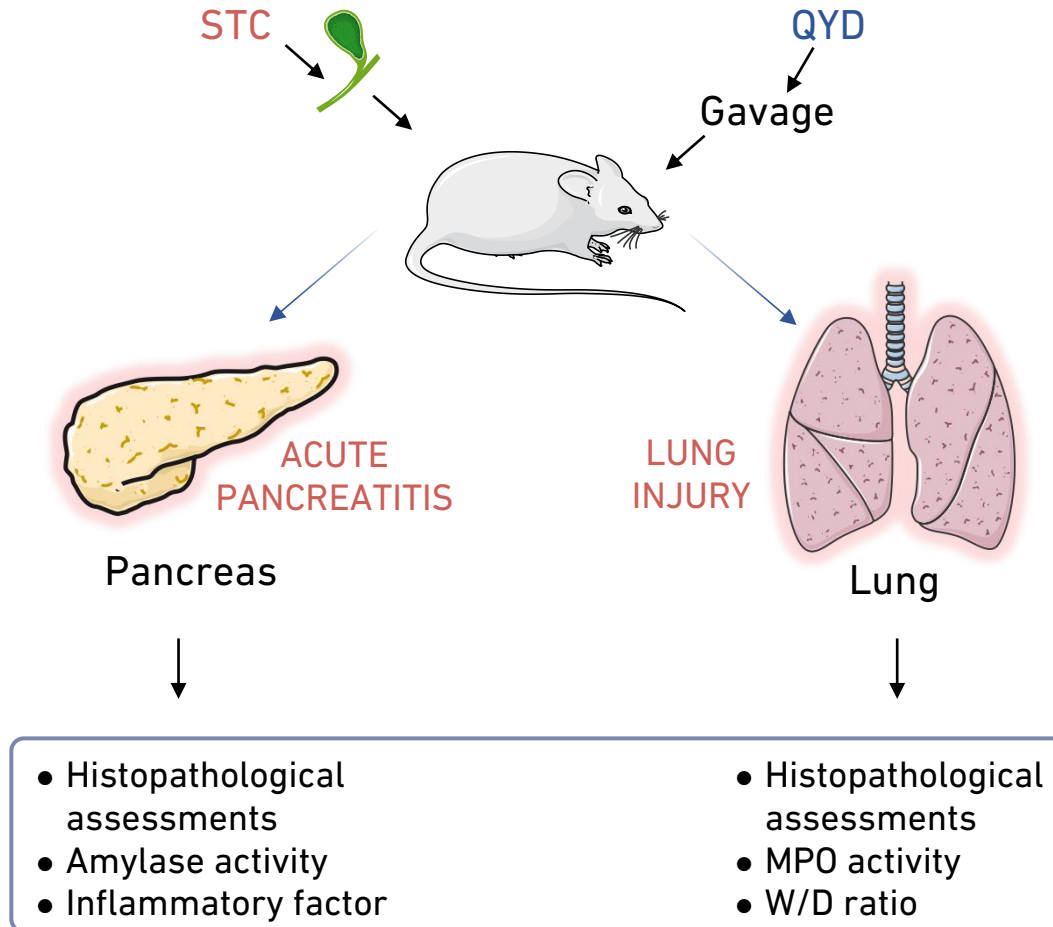

## QYD mode of action

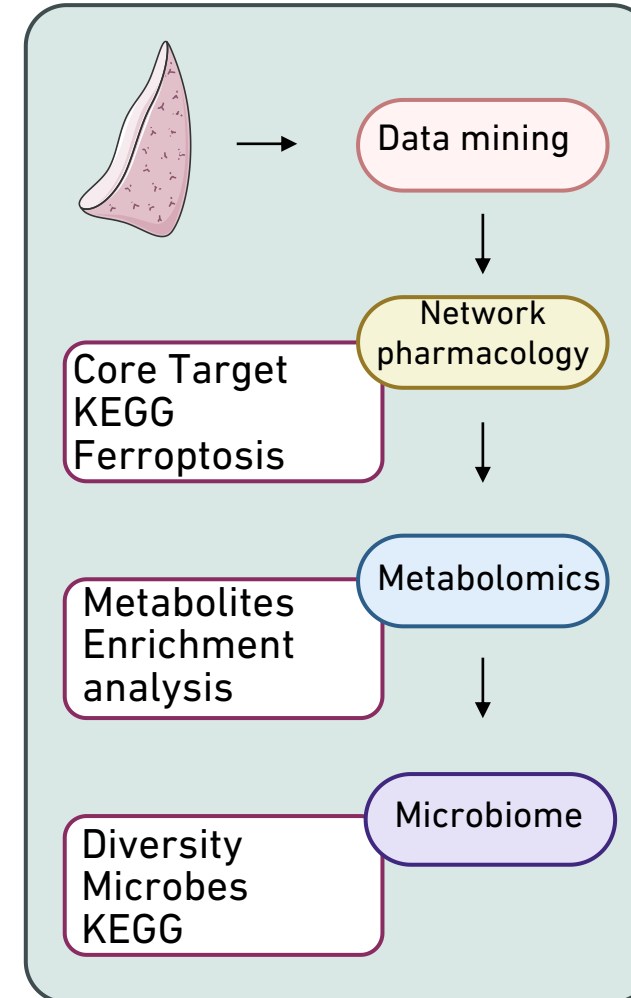

Supplement: Supplementary Materials — Supplementary File S1: 225 ingredients and 514 potential targets for QYD. Supplementary File S2: the sequences of the primers for qRT-PCR. Supplementary File S3: details of the analytical conditions and data preprocessing for mass spectrum. Supplementary File S4: the CDOCKER interaction energy of all ingredients and proteins. Supplementary File S5: apoptosis in the lung tissue of each group of rats. Supplementary File S6: expression of ferroptosis-related proteins in lung tissue of rats in each group. Supplementary File S7: expression of 8-OHdG in lung tissue of rats in each group. Supplementary File S8: effects of QYD and/or erastin on lung tissue damage and inflammation in SAP rats. Supplementary File S9: effect of QYD on the Shannon index, Simpson index, and Chao1 index of SAP rats. Graphical abstract: protective mechanism of QYD in SAP-associated ALI rat model. [file 5827613.f1.zip › Graphical abstract.pdf]
